# New insights into the ectoparasite fauna of bats (Phyllostomidae and Vespertilionidae) in the Baja California Peninsula, Mexico

**DOI:** 10.1007/s00436-025-08568-z

**Published:** 2025-11-25

**Authors:** Aimée I. Del Río-Trujillo, Juan B. Morales-Malacara, Aldo A. Guevara-Carrizales, Martín Y. Cabrera-Garrido, F. Sara Ceccarelli, Andrés Martínez-Aquino

**Affiliations:** 1 Laboratorio de Biología Evolutiva de Parásitos, Facultad de Ciencias, Universidad Autónoma de Baja California, Carretera Transpeninsular Ensenada-Tijuana No. 3917, Colonia Playitas, Ensenada, Baja California 22860 México; 2 Laboratorio de Espeleobiología y Acarología, Unidad Multidisciplinaria de Docencia e Investigación, Facultad de Ciencias, Universidad Nacional Autónoma de México, campus Juriquilla, Boulevard Juriquilla 3001, Querétaro, Querétaro 76230 México; 3 Colección de Vertebrados, Facultad de Ciencias, Universidad Autónoma de Baja California, Carretera Transpeninsular Ensenada-Tijuana No. 3917, Colonia Playitas, Ensenada, Baja California 22860 México; 4 Laboratorio de Macroecología Evolutiva, Instituto de Ecología, A.C., Red de Biología Evolutiva, 91070 Xalapa, Veracruz, México; 5 Departamento de Biología de la Conservación, Centro de Investigación Científica y Educación Superior de Ensenada, Carretera Ensenada-Tijuana No. 3918, Zona Playitas, Ensenada, Baja California 22860 México

**Keywords:** Acari, Baja California, Cimicidae, Ectoparasites, Nycteribiidae, Streblidae

## Abstract

Bat ectoparasites are an integral part of the wildlife and of host-parasite interactions in the Baja California Peninsula (BCP), Mexico, but the study of these ectoparasites is scarce in this area. In this study the ectoparasite faunal composition of the bat families Phyllostomidae and Vespertilionidae was determined and the first ectoparasitic checklist from BCP was developed. Thirteen bat species (Phyllostomidae, N = 1, Vespertilionidae, N = 12) from the northern and central region of the BCP were analysed. The checklist is presented in two tables, a parasite-host list and a host-parasite list. Twenty-three ectoparasite taxa were recorded, belonging to the five orders Ixodida (Argasidae), Mesostigmata (Macronyssidae, Spinturnicidae), Trombidiformes (Trombiculidae, Leeuwenhoekiidae, Myobiidae), Diptera (Nycteribiidae, Streblidae), and Hemiptera (Cimicidae), from nine bat species: *Leptonycteris yerbabuenae, Antrozous pallidus, Corynorhinus townsendii, Eptesicus fuscus, Myotis californicus, M. ciliolabrum, M. evotis, M. vivesi* and *M. volans*. Six taxa of Acari are new records for BCP and Mexico, i.e., *Albeckia albecki*, *Acanthophthirius caudatus eptesicus*, *Acanthophthirius* sp. 1, *Acanthophthirius* sp. 2, Trombiculidae Gen. sp. 1 and *Steatonyssus occidentalis*. Ectoparasites of Vespertilionidae likely share Nearctic affinities in relation with their hosts’ distribution, as ectoparasites of Phyllostomidae do with Neotropical affinities. This study is pioneer in the construction of baseline data of bat ectoparasite diversity in the BCP; due to lack of information, it is necessary to continue the studies of ectoparasites for bat species of the families Emballonuridae, Molossidae, Mormoopidae, and Natalidae.

**Supplementary information:**

The online version contains supplementary material available at 10.1007/s00436-025-08568-z.

## Introduction

The study and characterization of wildlife composition tends to focus on conspicuous groups, neglecting the parasite component and host-parasite interactions. Accounting for ectoparasite species diversity and host-parasite interactions is key for assembling baseline data for a more comprehensive checklist of faunal diversity; however, the lack of data can limit this kind of evaluation in understudied regions. Bat ectoparasites are hematophagous and/or histiophagous arthropods primarily represented by ticks (Ixodida), mites (Acari), bat flies (Nycteribiidae and Streblidae) and bat bugs (Cimicidae). They present host specificity that can range from polixenous (species that associate to hosts within the same order), oligoxenous (that associate within a family of hosts), stenoxenous (that associate within a host genus), and monoxenous (species are restricted to one host species) (Colín-Martínez et al. 2018).

The Baja California Peninsula (BCP), Mexico, comprising the states of Baja California (BC) and Baja California Sur (BCS), remains one of the least studied regions in Mexico regarding bat ectoparasites (Whitaker and Morales-Malacara 2005). Eight bat families are reported for Mexico (Ramírez-Pulido et al. 2014) with around 145 species, 20 being endemic (Sil-Berra et al. 2022; Garbino et al. 2024; López-Cuamatzi et al. 2024). Twenty-five bat species are reported in the BCP, belonging to the families Emballonuridae, Molossidae, Mormoopidae, Natalidae, Phyllostomidae, and Vespertilionidae, the last two with three and 15 species, respectively (Medellín et al. 2008; Ramírez-Pulido et al. 2014; Simmons and Cirranello 2025).

While over 70 bat ectoparasite species have been recorded for the rest of Mexico (Whitaker and Morales-Malacara 2005), records of bat ectoparasites in the BCP have been scarce and sporadic, with a total of nine species reported between 1924 and 1965 from one study per decade (Ferris 1924; Scott 1939; Guimaraes and D’andretta 1956; Kohls et al. 1965). Most of these studies were carried out on the islands of the Gulf of California (Ángel de la Guarda Island, Partida Island, Patos Island, Pescadero Island, Pond Island), which include punctual records (Guimaraes and D’andretta 1956), and taxonomic descriptions (Scott 1939; Kohls et al. 1965). One such taxonomic study was carried out by Uchikawa and Baker (1993) and the ectoparasites collected by Otálora-Ardila et al. (2022) were not identified, remaining as observations. In the most recent effort to characterize this diversity, Najera-Cortazar et al. (2023), using molecular data, detected one species and seven lineages of mites (i.e., Argasidae) and six species and 14 lineages of insects (i.e., Cimicidae, Nycteribiidae and Streblidae) of bat ectoparasites along the BCP. However, unfortunately their study did not complement their molecular analyses based on morphological identifications, and to date it is unknown whether there are reference specimens from the BCP region in any scientific collection to link to findings based on molecular data.

The main aim of this study was to establish baseline data of bat ectoparasites from the BCP to provide new insights that will serve as a foundation for future ectoparasitological research, by 1. determining with a morphological approach the bat ectoparasite species diversity found on Phyllostomidae and Vespertilionidae of the northern and central region of the BCP, and 2. constructing a checklist of host-parasite interactions.

## Methods

### Collection of bats

**Fig. 1:**
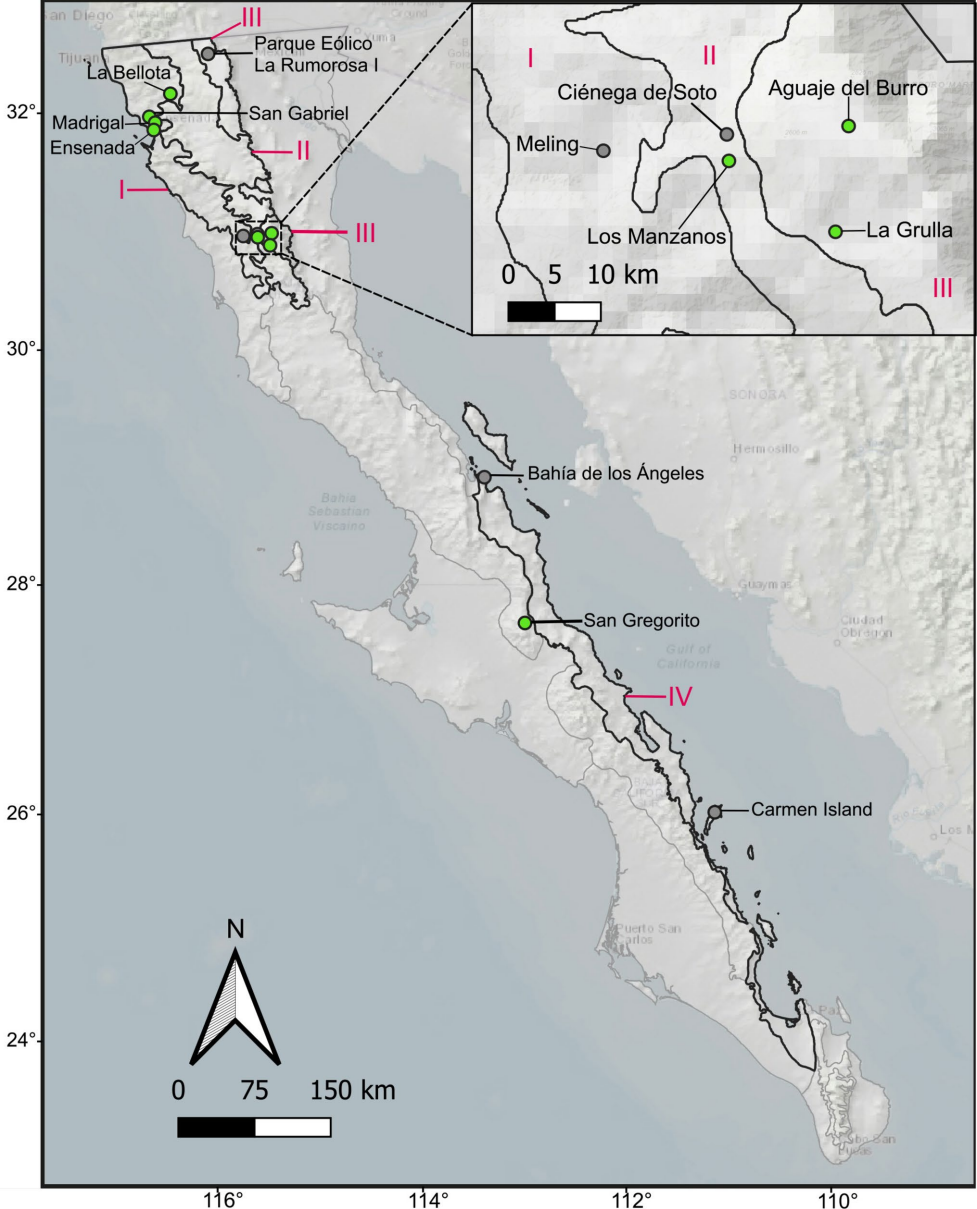
Map of original bat sampling localities (green) and sampled sites of bat ectoparasite specimens from CVUABC (grey). Ecoregions with ectoparasite records in this study are shown in bold lines. Ecoregions following González-Abraham et al. (2010). I: Coastal Sage Scrub. II: Chaparral. III: Sierras de Juárez y San Pedro Mártir. IV: Central Gulf Coast. Map made in QGIS v 3.30.2-’s-Hertogenbosch (QGIS.org 2023)

Bat sampling was carried out from April 2022 to June 2023 on eight localities distributed in four ecoregions in the states of BC and BCS, Mexico (Fig. 1, Table 1). Mist nets of 6 m or 12 m long × 2 m tall were installed over or near bodies of water in each site and were checked every hour from sunset, between 17:00–19:00 h, to 23:00 h. Each bat was individually stored in clean cloth bags to avoid host cross-contamination. Bats were identified in the field and lab using field guides (Álvarez et al. 1994; Medellín et al. 2008) and checked for sex and reproductive condition. Sampling was carried out under the permit SPARN/DGVS/04102/22 issued by Secretaría de Medio Ambiente y Recursos Naturales (SEMARNAT). Bat voucher specimens were deposited in the CVUABC (Table 1).

**Table 1:** Localities (from north to south) in the Baja California Peninsula, Mexico, where Phyllostomidae and Vespertilionidae bats were analysed in this study. BC = Baja California; BCS = Baja California Sur. * = sampled sites of bat ectoparasite specimens from Colección de Vertebrados Facultad de Ciencias, Universidad Autónoma de Baja California (CVUABC); ** = original bat sampling localities during this study; ―: No ectoparasites found. NH = number of hosts by bats species examined per sampled site and voucher number CVUABC

Locality	Municipality, State	Locality acronym	Coordinates	NH (No. CVUABC)	Sampling (month and year)
*Parque Eólico La Rumorosa I	Tecate, BC	Rumo	32.4905, −116.0885	3 *Antrozous pallidus* (released)	July 2009
				2 *Myotis ciliolabrum* (released)	July 2009
**La Bellota	Ensenada, BC	Bell	32.1600, −116.4571	1 *Corynorhinus townsendii* (1266)	May 2023
**San Gabriel	Ensenada, BC	Gabr	31.9687, −116.6630	1 *Myotis evotis* (1254)	April 2022
				1 *Lasiurus frantzii* (1239)	November 2022
				1 *Myotis ciliolabrum* (1256)	February 2023
**Madrigal	Ensenada, BC	―	31.9196, −116.6053	1 *Lasiurus frantzii* (1247)	February 2023
				4 *Lasiurus cinereus* (1248–1251)	February 2023
				2 *Lasiurus xanthinus* (1252,1253)	February 2023
**Ensenada	Ensenada, BC	―	31.8591, −116.6194	1 *Lasiurus xanthinus* (1189)	September 2022
**Aguaje del Burro	Ensenada, BC	Agbu	30.9958, −115.4663	1 *Myotis evotis* (1265)	April 2023
				2 *Eptesicus fuscus* (1267, 1268)	May 2023
*Ciénega de Soto	San Quintín, BC	Soto	30.9881, −115.6051	1 *Eptesicus fuscus* (released)	June 2012
				3 *Myotis evotis* (released)	June 2012
				4 *Myotis volans* (released)	June 2012
*Meling	San Quintín, BC	Meli	30.9722, −115.7441	1 *Antrozous pallidus* (1264)	April 2019
**Los Manzanos	San Quintín, BC	Manz	30.9623, −115.6020	6 *Myotis californicus* (1302–1307)	June 2023
**La Grulla	Ensenada, BC	―	30.8939, −115.4819	1 *Lasiurus cinereus* (1193)	October 2022
*Bahía de los Ángeles	San Quintín, BC	Bahi	28.9243, −113.3848	2 *Myotis vivesi* (0818, 0820)	July 2008
**San Gregorito	Mulegé, BCS	―	27.6665, −112.9878	1 *Leptonycteris yerbabuenae* (1168)	April 2022
				1 *Parastrellus hesperus* (1159)	April 2022
				1 *Antrozous pallidus* (1263)	April 2022
*Carmen Island	Loreto, BCS	Isca	26.0113, −111.1309	11 *Leptonycteris yerbabuenae* (1173, 1175, 1236–1238, 1257–1262)	March 2021

In addition to field sampling, bat and ectoparasite specimens from five other localities stored in the Colección de Vertebrados, Facultad de Ciencias, Universidad Autónoma de Baja California (CVUABC) were also examined. These included frozen bats (i.e., *Leptonycteris yerbabuenae* Martínez and Villa, 1940 and *Antrozous pallidus* (Le Conte, 1856); and ectoparasites stored in 70% ethanol vials from bat species *A. pallidus*, *Eptesicus fuscus* (Palisot de Beauvois, 1796), *Myotis ciliolabrum* (Merriam, 1886), *Myotis vivesi* Menegaux, 1901 and *Myotis volans* (H. Allen, 1866) (previously mentioned bat specimens were captured and released in the field).

### Ectoparasite collection and determination

Captured bats were euthanized using veterinary use pentobarbital sodium (Sikes and Animal Care and Use Committee of the American Society of Mammalogists 2016) in the Laboratorio de Biología Evolutiva de Parásitos (BEP), FC-UABC, and both fur and naked skin were carefully examined under dissecting microscope Zeiss Stemi 2000-C (Göttingen, Niedersachen, Germany). Ectoparasites were removed using entomological forceps and fine brushes (number 10/0). Mounting and taxonomic determination were done in Laboratorio de Espeleobiología y Acarología, Unidad Multidisciplinaria de Docencia e Investigación, Facultad de Ciencias, Universidad Nacional Autónoma de México, campus Juriquilla, Querétaro, Mexico (LEA-UMDI-FC-UNAM). Ticks and mites were treated with lactophenol for ten days to two weeks and mounted on slides with Hoyer’s medium, then kept on a Thermo Scientific heating plate (Langenselbold, Germany) at 45 °C for one week to improve clearing and remove all bubbles. Ectoparasitic insects were stored in vials with 70% ethanol (ticks too bulky to be mounted were also stored in 70% ethanol). Each preparation and vials were labelled with the individual(s) ectoparasite taxonomic determination (family, species, sex, who determined it taxonomically, decimal number of preparation if slide) and host information (bat species, sex, date of collection, locality, collector, voucher number).

Identification and taxonomical contrasting of the ectoparasite taxa was based on Kohls et al. (1965), Vercammen-Grandjean and Watkins (1966), Radovsky (1967), Fain and Whitaker (1976), Brennan and Goff (1977), Guzmán-Cornejo et al. (2019), Herrera-Mares et al. (2021) and Morales-Malacara and López-Ortega (2023) for mites; Wenzel et al. (1966), Theodor (1967) and Wenzel (1976) for bat flies; and Ueshima (1968) for bat bugs.

Photographs of the idiosoma and key diagnostic characters of all mite taxa were taken using a differential interference contrast (DIC) Zeiss Axio Imager.A2 microscope (Göttingen, Niedersachen, Germany) with AxioCam MRc and software AxioVision 4.8.2 (Göttingen, Niedersachen, Germany). Photographs of ectoparasitic insect taxa were taken using a Nikon SMZ745T stereoscope (Grand River Avenue, Brighton, Michigan) with Lumenera Infinity 1 camera and software Infinity Analyze 7.1.1 (Ottawa, Ontario, Canada).

Voucher ectoparasite specimens were deposited in Museo de Artrópodos de Baja California (MABC), housed at the Centro de Investigación Científica y de Educación Superior de Ensenada (CICESE), Ensenada, Baja California, Mexico, and the Morales-Malacara Collection (MM), housed at the LEA-UMDI-FC-UNAM. Host specificity information was compiled according to Colín-Martínez et al. (2018) (when not found, host specificity was classified to the level of ectoparasite-host relationship observed).

## Results

### Ectoparasite records

Of 52 individual bats belonging to 13 species (of the families Phyllostomidae, N = 1 and Vespertilionidae, N = 12), 473 individual ectoparasites were collected from 38 infested bats, including field sampling and collection specimens. This corresponds to an infestation rate of 73%. Twenty-three ectoparasite taxa were determined, belonging to six mite families (Argasidae, Macronyssidae, Spinturnicidae, Trombiculidae, Leeuwenhoekiidae, and Myobiidae), bat flies Nycteribiidae and Streblidae, and one species of bat bug belonging to the family Cimicidae (Tables 2 and 3; Supplementary Table S1). The nine families can be further reduced to three main groups (henceforth referred to as “ectoparasite groups”) which are mites (subclass Acari), flies (order Diptera) and bugs (order Hemiptera). Bats of the species *Lasiurus cinereus* (Palisot de Beauvois, 1796), *Lasiurus frantzii* (Peters, 1871), *Lasiurus xanthinus* Thomas, 1897 and *Parastrellus hesperus* (H. Allen, 1864) were not infested.

**Table 2:** Ectoparasite-host list of Phyllostomidae and Vespertilionidae bats in the Baja California Peninsula, Mexico. List of taxa according to Krantz and Walter (2009). Superscript shows the ectoparasite stage of development as follows: L = Larvae; N = Nymph; PN = Protonymph; DN = Deutonymph; DN♀ = Female deutonymph; DN♂ = Male deutonymph; DN to ♀ = Deutonymph to female; DN to ♂ = Deutonymph to male; ♀j = Juvenile female; ♀ = Female; ♂ = Male. Infestation site(s) (IS) are mentioned as follows; A = armpit; D = dorsal part of the body; E = ear; F = forearm; H = head; U = uropatagium; V = ventral part of the body; Wd = dorsal side of wings; Wv = ventral side of wings; # = ectoparasite seen running along the body; * = data not available. **–** = specimen(s) not stored in respective collection. Locality acronyms in Table 1. MM (UABC): voucher numbers of individuals deposited at the Morales-Malacara Collection housed at the LEA-UMDI-FC-UNAM; MABC: voucher numbers of individuals deposited at the Museo de Artrópodos de Baja California, CICESE

Ectoparasite taxa	Host	IS	Locality	MM (UABC)/MABC
Acari				
Argasidae Koch, 1844				
*Ornithodoros dyeri*^L^ Coley and Kohls, 1940	*Leptonycteris yerbabuenae*	*	Isca	MM (UABC): 1175.3–1175.5, 1175.7, 1175.8, 1175.10–1175.13/MABC: MABC-Ar-01019, MABC-Ar-01020
*Ornithodoros* sp. ^L^	*Antrozous pallidus*	*	Rumo	MM (UABC): ANPA02.1-ANPA02.4/MABC: MABC-Ar-01015, MABC-Ar-01016
Macronyssidae Oudemans, 1936				
*Cryptonyssus desultorius*^PN, ♀, ♂^ Radovsky 1966	*Myotis californicus*	U, Wv	Manz	MM (UABC): 1302.3–1302.5, 1304.15–1304.18, 1304.20–1304.22, 1304.30–1304.32, 1306.3 and 1306.6/MABC: MABC-Ar-01023, MABC-Ar-01024, MABC-Ar-01030
*Macronyssus crosbyi*^PN, DN, DN to ♀, DN to ♂, ♀, ♂^ (Ewing and Stover, 1915)	*Eptesicus fuscus*	*	Soto	MM (UABC): EPFU01.4/–
	*Myotis californicus*	D, U, V, Wd, Wv	Manz	MM (UABC): 1303.3–1303.20, 1304.1–1304.7, 1304.9, 1304.11–1304.14, 1304.19, 1304.23, 1304.24, 1305.1–1305.19, 1305.25, 1306.2, 1306.4, 1306.5, 1306.7, 1307.2–1307.6/MABC: MABC-Ar-01025-MABC-Ar-01028
*Macronyssus unidens*^PN^ Radovsky 1967	*Eptesicus fuscus*	V, Wd, Wv	Agbu	MM (UABC): 1268.1, 1268.3–1268.5, 1268.11, 1268.15/MABC: MABC-Ar-01031
*Steatonyssus antrozoi*^PN^ Radovsky and Furman 1963	*Antrozous pallidus*	V, Wd, Wv	Meli	MM (UABC): 1264.1–1264.3, 1264.5–1264.36/MABC: MABC-Ar-01033
	*Eptesicus fuscus*	Wd, Wv	Agbu	MM (UABC): 1268.10, 1268.12, 1268.17/–
	*Leptonycteris yerbabuenae*	Wd, Wv	Isca	MM (UABC): 1257.1/–
*Steatonyssus occidentalis*^♀, ♂^ (Ewing, 1933)	*Eptesicus fuscus*	V	Agbu	MM (UABC): 1267.17, 1267.18, 1267.20–1267.22/MABC: MABC-Ar-01032
Spinturnicidae Oudemans, 1902				
*Periglischrus paracaligus*^PN, DN♀, DN♂, ♀j, ♀, ♂^ Herrin and Tipton, 1975	*Leptonycteris yerbabuenae*	D, H, V, Wd, Wv	Isca	MM (UABC): 1175.1, 1175.2, 1238.1, 1238.3–1238.7, 1257.2–1257.7, 1260.1, 1261.1, 1261.3–1261.6, 1261.8–1261.14/MABC: MABC-Ar-01017, MABC-Ar-01018, MABC-Ar-01021, MABC-Ar-01022
*Spinturnix mexicana*^PN, ♀, ♂^ Rudnick 1960	*Myotis vivesi*	*	Bahi	MM (UABC): 0818.2, 0818.3, 0818.5–0818.9, 0820.2–0820.9/MABC: MABC-Ar-01034-MABC-Ar-01036
Trombiculidae Ewing, 1929				
Trombiculidae Gen. sp. 1 ^L^	*Eptesicus fuscus*	V	Agbu	MM (UABC): 1268.7/–
	*Myotis californicus*	E	Manz	MM (UABC): 1302.1, 1302.6–1302.9, 1302.12–1302.15, 1302.18–1302.21, 1302.27, 1302.39, 1302.40, 1302.43, 1302.45, 1302.46, 1302.48, 1302.50–1302.143/–
	*Myotis evotis*	*	Agbu	MM (UABC): MYEVMI03.1/–
*Euschoengastia* sp. ^L^	*Eptesicus fuscus*	V	Agbu	MM (UABC): 1268.9/–
*Loomisia* sp. ^L^	*Corynorhinus townsendii*	E	Bell	MM (UABC): 1266.1–1266.3/–
Leeuwenhoekiidae Womersley, 1945				
*Albeckia albecki*^L^ Vercammen-Grandjean and Watkins 1966	*Eptesicus fuscus*	H, U, V, Wd, Wv	Agbu	MM (UABC): 1267.1–1267.11, 1267.13- 1267.15, 1268.6/–
	*Myotis californicus*	E	Manz	MM (UABC): 1302.10, 1302.11, 1302.16, 1302.22–1302.26, 1302.28–1302.31, 1302.33–1302.38, 1302.41, 1302.42, 1302.44, 1302.47–1302.49/MABC: MABC-Ar-01029
Myobiidae Mégnin, 1877				
*Acanthophthirius caudatus eptesicus*^N, ♀^ Fain and Whitaker 1976	*Eptesicus fuscus*	H, V	Agbu	MM (UABC): 1267.16, 1268.8, 1268.13, 1268.14/–
*Acanthophthirius* sp. 1 ^N, ♀^	*Myotis ciliolabrum*	H	Gabr	MM (UABC): 1256.2–1256.7/–
	*Myotis evotis*	H	Agbu	MM (UABC): 1265.1, 1265.2/–
*Acanthophthirius* sp. 2 ^♂^	*Myotis californicus*	H	Manz	MM (UABC): 1305.26/–
Diptera				
Nycteribiidae Samouelle, 1819				
*Basilia antrozoi*^♀, ♂^ (Townsend, 1893)	*Antrozous pallidus*	*, A	Rumo	MM (UABC): ANPA03, 1264/–
*Basilia corynorhini*^♂^ (Ferris, 1916)	*Corynorhinus townsendii*	#	Bell	MM (UABC): 1266/MABC: MABC-Ar-01014
*Basilia forcipata*^♀, ♂^ Ferris 1924	*Myotis californicus*	#	Manz	MM (UABC): 1303/–
	*Myotis evotis*	*	Soto	–/MABC: MABC-xx-00115, MABC-xx-00116
	*Myotis volans*	*	Soto	MM (UABC): MYVO01, MYVO04/MABC: MABC-xx-00111-MABC-xx-00114
*Basilia pizonychus*^♀, ♂^ Scott 1939	*Myotis vivesi*	*	Bahi	MM (UABC): 0818/–
Streblidae Kolenati, 1863				
*Nycterophilia coxata*^♀, ♂^ Ferris, 1916	*Leptonycteris yerbabuenae*	*	Isca	MM (UABC): 1238/MABC: MABC-Ar-01003, MABC-Ar-01005-MABC-Ar-01010, MABC-Ar-01012
*Trichobius sphaeronotus*^♀, ♂^ Jobling, 1939	*Leptonycteris yerbabuenae*	*	Isca	MM (UABC): 1175, 1261/MABC: MABC-Ar-01004, MABC-Ar-01011, MABC-Ar-01013
Hemiptera				
Cimicidae Latreille, 1802				
*Cimex pilosellus*^♀, ♂^ (Horvath, 1910)	*Myotis californicus*	F	Manz	–/MABC: MABC-xx-00118-MABC-xx-00121
	*Myotis ciliolabrum*	*	Rumo	MM (UABC): MYME01, MYME02/MABC: MABC-xx-00109, MABC-xx-00110

**Table 3:** Host-ectoparasite list. Superscript shows the ectoparasite stage of development as follows: L = Larvae; N = Nymph; PN = Protonymph; DN = Deutonymph; DN♀ = Female deutonymph; DN♂ = Male deutonymph; DN to ♀ = Deutonymph to female; DN to ♂ = Deutonymph to male; ♀j = Juvenile female; ♀ = Female; ♂ = Male

Host	Ectoparasite taxa
Phyllostomidae Gray, 1825	
*Leptonycteris yerbabuenae* Martínez and Villa, 1940	Acari
	*Ornithodoros dyeri*^L^
	*Steatonyssus antrozoi*^PN^
	*Periglischrus paracaligus*^PN, DN♀, DN♂, ♀j, ♀, ♂^
	Diptera
	*Nycterophilia coxata*^♀, ♂^
	*Trichobius sphaeronotus*^♀, ♂^
Vespertilionidae Gray, 1821	
*Antrozous pallidus* (Le Conte, 1856)	Acari
	*Ornithodoros* sp. ^L^
	*Steatonyssus antrozoi*^PN^
	Diptera
	*Basilia antrozoi*^♀, ♂^
*Corynorhinus townsendii* (Cooper, 1837)	Acari
	*Loomisia* sp. ^L^
	Diptera
	*Basilia corynorhini*^♂^
*Eptesicus fuscus* (Palisot de Beauvois, 1796)	Acari
	*Macronyssus crosbyi*^PN^
	*Macronyssus unidens*^PN^
	*Steatonyssus antrozoi*^PN^
	*Steatonyssus occidentalis*^♀, ♂^
	Trombiculidae Gen. sp. 1 ^L^
	*Euschoengastia* sp. ^L^
	*Albeckia albecki*^L^
	*Acanthophthirius caudatus eptesicus*^N, ♀^
*Myotis californicus* (Audubon and Bachman, 1842)	Acari
	*Cryptonyssus desultorius*^PN, ♀, ♂^
	*Macronyssus crosbyi*^PN, DN, DN to ♀, DN to ♂, ♀, ♂^
	Trombiculidae Gen. sp. 1 ^L^
	*Albeckia albecki*^L^
	*Acanthophthirius* sp. 2 ^♂^
	Diptera
	*Basilia forcipata*^♀^
	Hemiptera
	*Cimex pilosellus*^♀, ♂^
*Myotis evotis* (H. Allen, 1864)	Acari
	Trombiculidae Gen. sp. 1 ^L^
	*Acanthophthirius* sp. 1 ^N, ♀^
	Diptera
	*Basilia forcipata*^♀^
*Myotis ciliolabrum* (Merriam, 1886)	Acari
	*Acanthophthirius* sp. 1 ^N, ♀^
	Hemiptera
	*Cimex pilosellus*^♀, ♂^
*Myotis vivesi* Menegaux, 1901	Acari
	*Spinturnix mexicana*^PN, ♀, ♂^
	Diptera
	*Basilia pizonychus*^♀, ♂^
*Myotis volans* (H. Allen, 1866)	Diptera
	*Basilia forcipata*^♀, ♂^

**Fig. 2:**
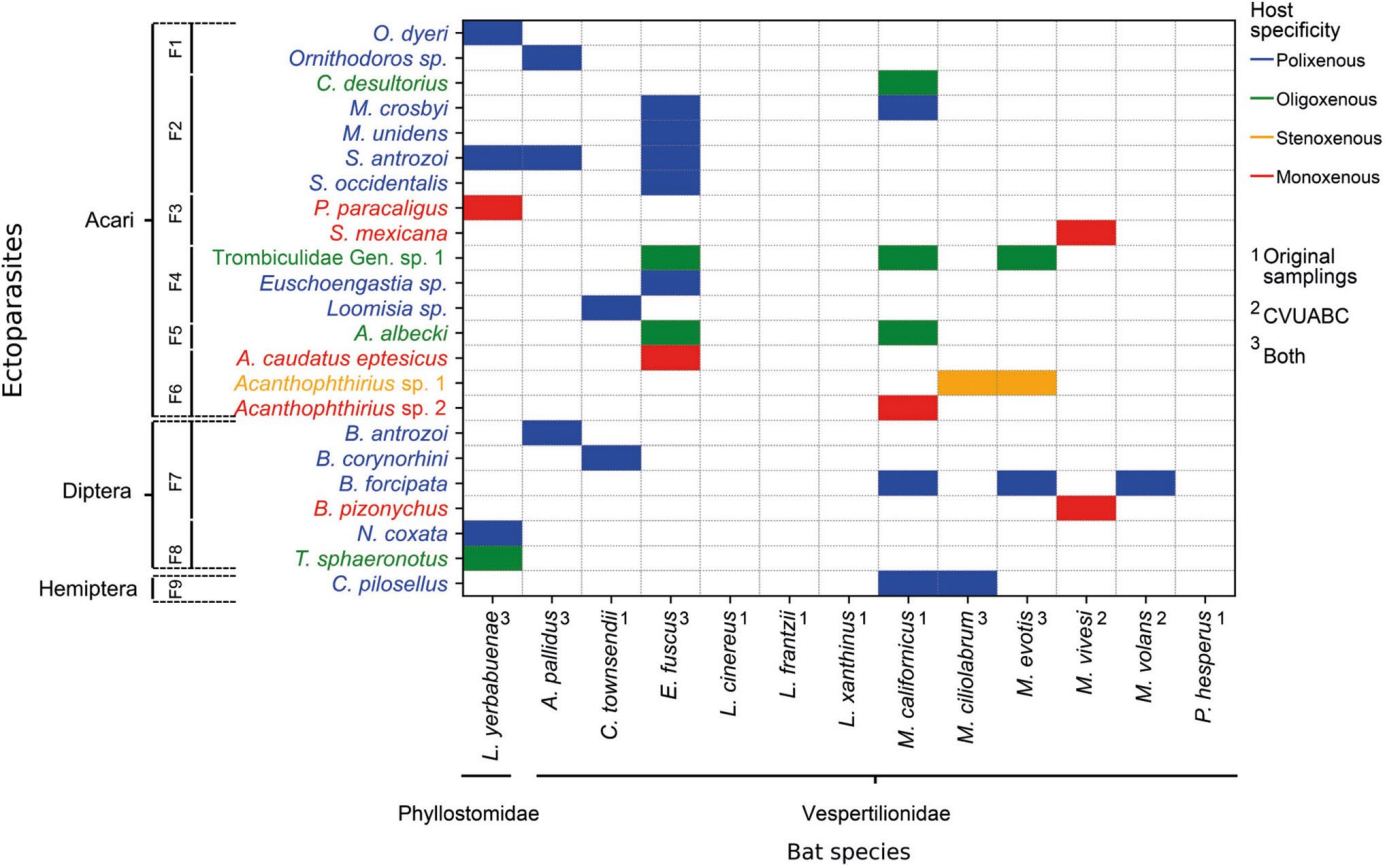
Presence/absence of ectoparasite taxa found on each bat host species and their host specificity level according to the colour code. Bat species are shown in alphabetical order. F1 = Argasidae. F2 = Macronyssidae. F3 = Spinturnicidae. F4 = Trombiculidae. F5 = Leeuwenhoekiidae. F6 = Myobiidae. F7 = Nycteribiidae. F8 = Streblidae. F9 = Cimicidae. Figure made in Python (3.10.15, https://www.python.org/) in Jupyter notebook (Version: 7.0.8, https://github.com/jupyter/notebook)

The highest number of ectoparasite species (eight) were found on *E. fuscus*, followed by *Myotis californicus* (Audubon and Bachman, 1842) (seven), *L. yerbabuenae* (five), *A. pallidus* and *Myotis evotis* (H. Allen, 1864) (three), *Corynorhinus townsendii* (Cooper, 1837), *M. ciliolabrum* and *M. vivesi* (two), and the lowest on *M. volans* (one). *Myotis californicus* was the only bat species infested by species from the three ectoparasite groups (Acari, Diptera, Hemiptera). Four levels of host specificity were found; in number of species the best represented was polixenous, followed by monoxenous (five in total, two found on *M. vivesi*), oligoxenous, and stenoxenous (one species, the least represented) (see Fig. 2).

**Fig. 3:**
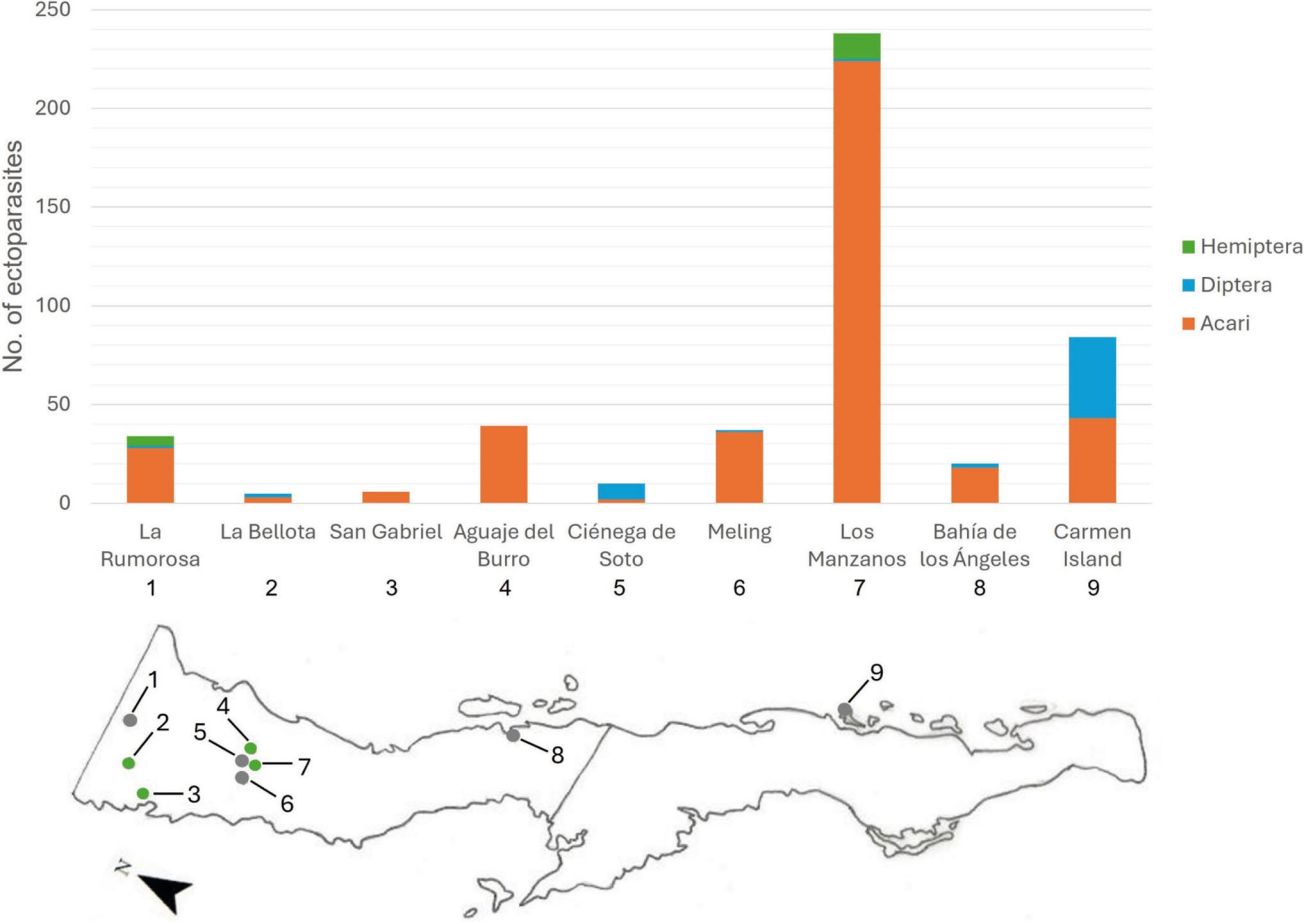
Latitudinal distribution of collected ectoparasites corresponding to their localities throughout the Baja California Peninsula. Numbers 1–9 indicate geographic position of each locality and dot colour indicates the origin of each ectoparasite group (green: original specimens, grey: CVUABC samples)

Ectoparasites were recorded from nine of the 13 localities and the total number of ectoparasites by locality and their latitudinal distribution is shown on Fig. 3. Aguaje del Burro and Los Manzanos presented seven species (the latter presenting the three ectoparasite groups), followed by Carmen Island with five, Ciénega de Soto and Parque Eólico La Rumorosa I with three, Bahía de los Ángeles, La Bellota and Meling with two, and San Gabriel with only one ectoparasite species.

### Mites and ectoparasitic insects

The mites *Steatonyssus antrozoi* Radovsky and Furman 1963 and Trombiculidae Gen sp. 1 were found in three bat species, having the main mite parasitic presence (infesting *A. pallidus*, *E. fuscus* and *L. yerbabuenae*, and *E. fuscus*, *M. californicus* and *M. evotis*, respectively). In contrast, *Euschoengastia* sp. and *Acanthophthirius* sp. 2 showed the least parasitic presence, both having one specimen found in their respective bat species (*E. fuscus* and *M. californicus*). The complete determination of *Ornithodoros* sp. specimens was not achieved due to the absence of the hypostome, which has taxonomic value.

For the insects, *Basilia forcipata* Ferris 1924 showed the main parasitic presence, found in three bats species (*M. californicus*, *M. evotis* and *M. volans*). All ectoparasitic insects were adults. The least insect parasitic presence was shared by *Basilia corynorhini* (Ferris, 1916), only two male flies infesting *C. townsendii*, and *Basilia pizonychus* Scott 1939, a female and male infesting *M. vivesi*. All collected specimens of *Cimex pilosellus* (Horvath, 1910) were found infesting the forearm of *M. californicus*. The mite *S. antrozoi* was the only ectoparasite found infesting both bat families.

### New records

The following ectoparasite taxa are recorded for the first time in the BCP. *Ornithodoros* sp. (on *A. pallidus*); *Albeckia albecki* Vercammen-Grandjean and Watkins 1966 (on *E. fuscus* and *M. californicus*); *Cryptonyssus desultorius* Radovsky 1966 (on *M. californicus*), *Macronyssus crosbyi* (Ewing and Stover 1915) (on *E. fuscus* and *M. californicus*); *Macronyssus unidens* Radovsky 1967 (on *E. fuscus*); *S. antrozoi* (on *A. pallidus*); *Steatonyssus occidentalis* (Ewing 1933) (on *E. fuscus*); *Acanthophthirius caudatus eptesicus* Fain and Whitaker 1976 (on *E. fuscus*); *Acanthophthirius* sp. 1 (on *M. ciliolabrum* and *M. evotis*); *Acanthophthirius* sp. 2 (on *M. californicus*); *Periglischrus paracaligus* Herrin & Tipton, 1975 (on *L. yerbabuenae*); *Spinturnix mexicana* Rudnick 1960 (on *M. vivesi*); Trombiculidae Gen. sp. 1 (on *E. fuscus*, *M. californicus* and *M. evotis*); *Euchoengastia* sp. (on *E. fuscus*); and *Loomisia* sp. (on *C. townsendii*); *B. corynorhini* (on *C. townsendii*); *B. forcipata* (on *M. californicus*, *M. evotis* and *M. volans*); and *C. pilosellus* (on *M. californicus* and *M. ciliolabrum*).

Five previously recorded ectoparasite species are re-confirmed records for the BCP based on our study. *Ornithodoros dyeri* Coley and Kohls, 1940, *Nycterophilia coxata* Ferris, 1916 and *Trichobius sphaeronotus* Jobling, 1939 (on *L. yerbabuenae*); *Basilia antrozoi* (Townsend, 1893) (on *A. pallidus*); and *B. pizonychus* (on *M. vivesi*).

## Discussion

### Ectoparasites of Phyllostomidae and Vespertilionidae

This study presents one of the first taxonomic approaches to bat ectoparasite diversity in the BCP. Previous studies report nine ectoparasite species (Ferris 1924; Scott 1939; Kohls et al. 1965; Radovsky 1966; Vercammen-Grandjean and Watkins 1966; Uchikawa and Baker 1993; Najera-Cortazar et al. 2023), while this study documents 13 additional mite taxa, two bat fly species, and one bat bug species, increasing the total taxonomic diversity to 25 ectoparasite taxa. The previous statement represents a 177% increase in species found throughout BCP. Spinturnicidae and Trombiculidae specimens are also recorded for the first time in this region. Mites *Acanthophthirius pizonixeos* Uchikawa and Baker 1993 (Uchikawa and Baker 1993) and *Ornithodoros kelleyi* (Cooley and Kohls, 1941) (Najera-Cortazar et al. 2023) were the only previously reported ectoparasites not found in this study.

Three bat species of the family Vespertilionidae (*C. townsendii*, *M. ciliolabrum*, and *M. evotis*) are recorded as hosting ectoparasites for the first time in the BCP, increasing the total number of species in this family with such records to 11 (Ferris 1924; Guimaraes and D’andretta 1956; Kohls et al. 1965; Radovsky 1966; Najera-Cortazar et al. 2023). No bats of the Phyllostomidae family present new ectoparasite records.

Concerning the nature of the CVUABC ectoparasites samples analysed in this study (from host species *L. yerbabuenae*, *A. pallidus*, *E. fuscus*, *M. ciliolabrum*, *M. evotis*, *M. vivesi*, and *M. volans*), the low ectoparasitic presence results must be considered with precaution because of sampling bias, e.g., these specimens were not collected for parasitological studies (Guevara-Carrizales, pers. comm.).

The absence of ectoparasites in *L. cinereus, L. frantzii*, *L. xanthinus*, and *P. hesperus* is likely influenced by the solitary and non-cave-roosting behaviours of these bat genera (Cross 1965; Shump and Shump 1982a, 1982b; Kurta and Lehr 1995), as well as the suppressive effect of winter months on ectoparasite reproduction. Ectoparasite records for *L. cinereus* indicate low parasitic intensity, a maximum of two ectoparasite individuals per bat for both mites (Whitaker and Easterla 1975; Whitaker and Morales-Malacara 2005) and bat flies (Graciolli et al. 2007). Polaco et al. (1994) reported a similar low parasitic intensity in *C. pilosellus* for *P. hesperus*. Cold temperatures further reduce reproduction and infestation rates during winter in temperate zones (Marshall 1982). Most *Lasiurus* individuals (seven out of ten) were captured in winter (February), potentially explaining the absence of ectoparasites. Current data on winter ectoparasite ecology of Vespertilionidae are from cave-roosting species (Reisen et al. 1976; Zahn and Rupp 2004; Lourenço and Palmeirim 2008). The lack of data on solitary bat species highlights a critical gap in the available information. This study suggests that low parasitic intensity, solitary behaviour, and winter season effects contribute to the lack of ectoparasites in *L. cinereus, L. frantzii* and *L. xanthinus*. For *P. hesperus*, this is likely due to low parasitic intensity, as the only sampled individual (captured in April), showed no infestation of bat bugs. The solitary habits of *C. townsendii* (Kunz and Martin 1982) also likely explain the low ectoparasite count found on the analysed bat individual (only three Trombiculidae mites), with previous records supporting this suggestion (Whitaker and Easterla 1975; Ritzi et al. 2001).

In this study, *E. fuscus* presented the highest mite taxa richness. Kurta and Baker (1990) mentioned the presence of 13 mite genera on *E. fuscus* throughout the Americas, including *Acanthophthirius*, *Euschoengastia*, *Macronyssus* and *Steatonyssus*. The ectoparasitic insect diversity observed on *M. californicus* was similar to that reported by Najera-Cortazar et al. (2023); i.e., two *Cimex* lineages and one Nycteribiidae lineage. All *M. californicus* individuals predominantly hosted *C. desultorius*, *M. crosbyi* and *C. pilosellus*, suggesting the possibility of shared shelter exposure to these ectoparasites.

### Ticks and mites

The mites *P. paracaligus, S. mexicana*, *A. caudatus eptesicus* are known monoxenous species (Fain and Whitaker 1976; Whitaker et al. 1983; Whitaker and Morales-Malacara 2005; Zamora-Mejías et al. 2020a), while *Acanthophthirius* sp. 2 is considered monoxenous in this study. Since *Acanthophthirius* sp. 2 is a new record for Mexico it suggests the possibility of identifying additional bat hosts. *Acanthophthirius* sp. 1 represents the only stenoxenous taxa, associated to *M. ciliolabrum* and *M. evotis*. Additional collections of *M. californicus*, *M. ciliolabrum* and *M. evotis* are needed to obtain adult females and males of these ectoparasites for detailed taxonomic determination (Morales-Malacara, pers. comm.). Oligoxenous species include *C. desultorius, S. antrozoi* and *A. albecki* (Whitaker and Wilson 1974; Whitaker et al. 1983; Zajkowska et al. 2018), although the presence of *S. antrozoi* on a bat of the Phyllostomidae family in this study suggests a polixenous specificity. Since Trombiculidae Gen. sp. 1 was found on *E. fuscus, M. californicus* and *M. evotis*, in this study it is tentatively considered an oligoxenous taxa. The remaining taxa exhibit polixenous specificity, which is also the predominant host specificity, i.e., *O. dyeri*, *Ornithodoros* sp. *M. crosbyi*, *M. unidens*, *S. occidentalis, Euschoengastia* sp. and *Loomisia* sp. (Whitaker and Morales-Malacara 2005). The previously mentioned taxa, excluding *M. crosbyi*, were found on only one bat species.

Mites were the most representative group by sampled sites in this study (nine out of 13), unlike in Najera-Cortazar et al. (2023) where bat flies predominated. Out of the 14 ecoregions found in the BCP (González-Abraham et al. 2010), seven localities fell within three ecoregions (Coastal Sage Scrub, Chaparral, Sierras de Juárez y San Pedro Mártir) could indicate a distribution pattern influenced by geographical proximity, while two sites (Bahía de los Ángeles and Carmen Island) belonged to a single ecoregion further south (Central Gulf Coast). Bat ectoparasite distribution depends on their host specificity (Dick and Patterson 2006; Durden 2006) and the range of distribution of their host species (e.g., Ceballos 2014). Since the ecoregions of Coastal Sage Scrub, Chaparral, and Sierras de Juárez y San Pedro Mártir are influenced by the Nearctic region, mite species *A. albecki*, *C. desultorius*, *M. unidens*, *Ornithodoros* sp., *S. antrozoi, S. mexicana* and *S. occidentalis* found in this study likely share a Nearctic affinity. This is supported by their Vespertilionidae hosts’ distribution (Ceballos 2014; Taylor and Tuttle 2019), and ectoparasitological records in the Nearctic (Rudnick 1960; Radovsky and Furman 1963; Radovsky 1966; Vercammen-Grandjean and Watkins 1966). *Ornithodoros dyeri* and *P. paracaligus*, which were found in the Central Gulf Coast ecoregion, likely share Neotropical affinity due to the seasonal migration of the *L. yerbabuenae* population on Carmen Island to the mainland (Frick et al. 2018). This migration promotes mite dispersal, aligning their presence with other Mexican maternity colonies (Zamora-Mejías et al. 2020a, b). Since the Central Gulf Coast ecoregion lies within the Mexican Nearctic, this likely Neotropical affinity of mites *O. dyeri* and *P. paracaligus* distinguish them from other mites found on the BCP.

### Ectoparasitic insects

Bats of the families Phyllostomidae and Vespertilionidae did not share ectoparasitic insect species. Species of *Basilia* showed a preference for Vespertilionidae even among polixenous species (*B. antrozoi, B. corynorhini*, and *B. forcipata*), paralleling the close relationship of Nycteribiidae-Vespertilionidae (Marshall 1982; Dick and Patterson 2006). *Nycterophilia coxata* and *T. sphaeronotus* were the only bat flies previously recorded in the BCP for *L. yerbabuenae* (Najera-Cortazar et al. 2023). The bat fly *B. pizonychus* is monoxenous to *M. vivesi* (Whitaker and Morales-Malacara 2005). The bat fly *T. sphaeronotus* was the only oligoxenous species, parasitising *L. yerbabuenae*, consistent with previous records (Hoffmann 1953; Ryckman 1956; Ramírez-Martínez and Tlapaya-Romero 2023). In this study, the predominant specificity level in insects was polixenous, showed by *B. antrozoi, B. corynorhini, B. forcipata*, *N. coxata,* and *C. pilosellus,* similar to previously reported (e.g., Ferris 1924; Guimaraes and D’andretta 1956; Whitaker and Easterla 1975; Polaco et al. 1994). *Basilia antrozoi* is commonly associated with *A. pallidus* (Graciolli et al. 2007), and in this study also was found in this host species.

Ectoparasitic insects were recorded in the four analysed ecoregions, but only *C. pilosellus* appeared in Sierras de Juárez y San Pedro Mártir. *Cimex* spp. and *Basilia* spp. show Nearctic affinities, consistent with their hosts’ range of distribution (Usinger 1966; Ceballos 2014; Taylor and Tuttle 2019) and locations of previous ectoparasite records within the Nearctic region (Ferris 1924; Polaco et al. 1994; Villegas-Guzmán et al. 2003, 2005). Streblidae species, on the other hand, likely show Neotropic affinities since they are linked to Phyllostomidae (Dick and Patterson 2006), a group mainly found in the Neotropics.

## Conclusion

Twenty-three bat ectoparasite taxa were determined, Phyllostomidae presenting five taxa (one monoxenous) and Vespertilionidae presenting 19 taxa (four monoxenous). Most of the first-time records correspond to mites. This contributes to the increase of bat ectoparasite biodiversity in the BCP to 25 taxa; however, it is necessary to continue the studies of ectoparasites due to the lack of information for bat species of the families Emballonuridae, Molossidae, Mormoopidae, and Natalidae. Additionally, a sampling design with standardized efforts throughout localities and seasons will allow for stronger conclusions regarding ectoparasite ecology and biology in BCP.

## Supplementary information

Below is the link to the electronic supplementary material.

Supplementary file 1 (DOCX 4.04 MB)

## Data Availability

No datasets were generated or analysed during the current study.
