# Supplementary material for: New insights into the ectoparasite fauna of bats (Phyllostomidae and Vespertilionidae) in the Baja California Peninsula, Mexico
Source: Parasitol Res. 2025 Nov 25;124(11):144. doi: 10.1007/s00436-025-08568-z (PMC12662873; doi:10.1007/s00436-025-08568-z)
Supplement: Supplementary file 1 — (DOCX 4.04 MB) [file 436_2025_8568_MOESM1_ESM.docx]

**Supplementary material**

**New insights into the ectoparasite fauna of bats (Phyllostomidae and Vespertilionidae) in the Baja California Peninsula, Mexico**

**Parasitology Research**

Aimée I. Del Río-Trujillo, Juan B. Morales-Malacara, Aldo A. Guevara-Carrizales, Martín Y. Cabrera-Garrido, F. Sara Ceccarelli and Andrés Martínez-Aquino*

*Corresponding author: [andres.martinez.aquino@uabc.edu.mx](mailto:andres.martinez.aquino@uabc.edu.mx).

Laboratorio de Biología Evolutiva de Parásitos, Facultad de Ciencias, Universidad Autónoma de Baja California, Carretera Transpeninsular Ensenada-Tijuana No. 3917, Colonia Playitas, 22860, Ensenada, Baja California, México.

**Supplementary Table S1**. Ectoparasite taxa photographs.

| **Acari** |
| --- |
| Argasidae Koch, 1844 |
| Figure S1. *Ornithodoros dyeri* Coley & Kohls, 1940  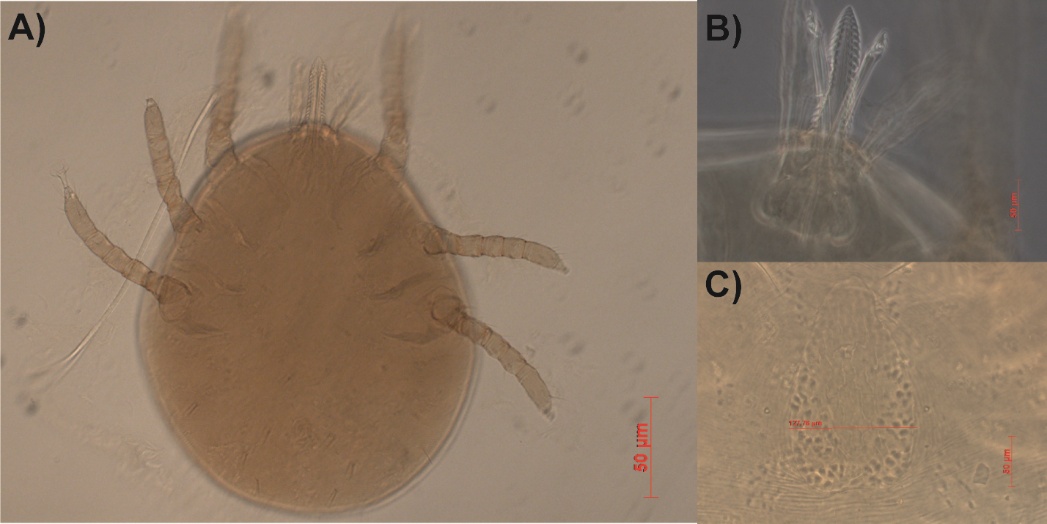  A) Idiosoma. B) Hypostome. C) Dorsal plate. |
| Figure S2. *Ornithodoros* sp.  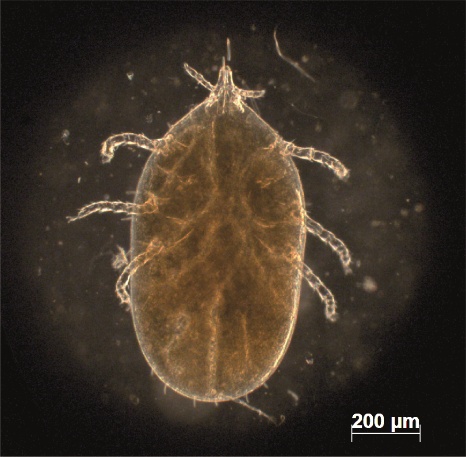  Idiosoma |
| Macronyssidae Oudemans, 1936 |
| Figure S3. *Cryptonyssus desultorius* Radovsky, 1966  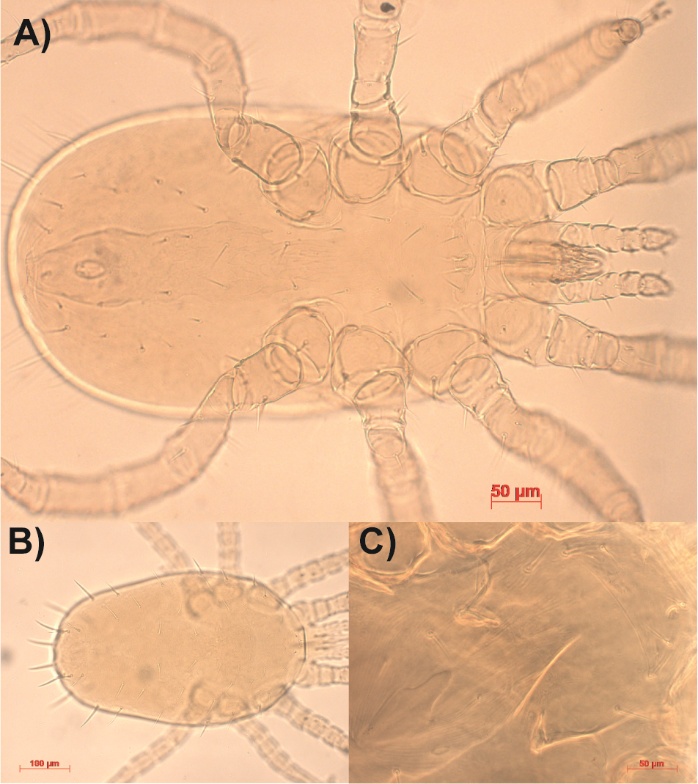  A) Idiosoma and holoventral plate (male). B) Dorsal plate (protonymph). C) Sternogenital plate (female). |
| Figure S4. *Macronyssus crosbyi* (Ewing & Stover, 1915)  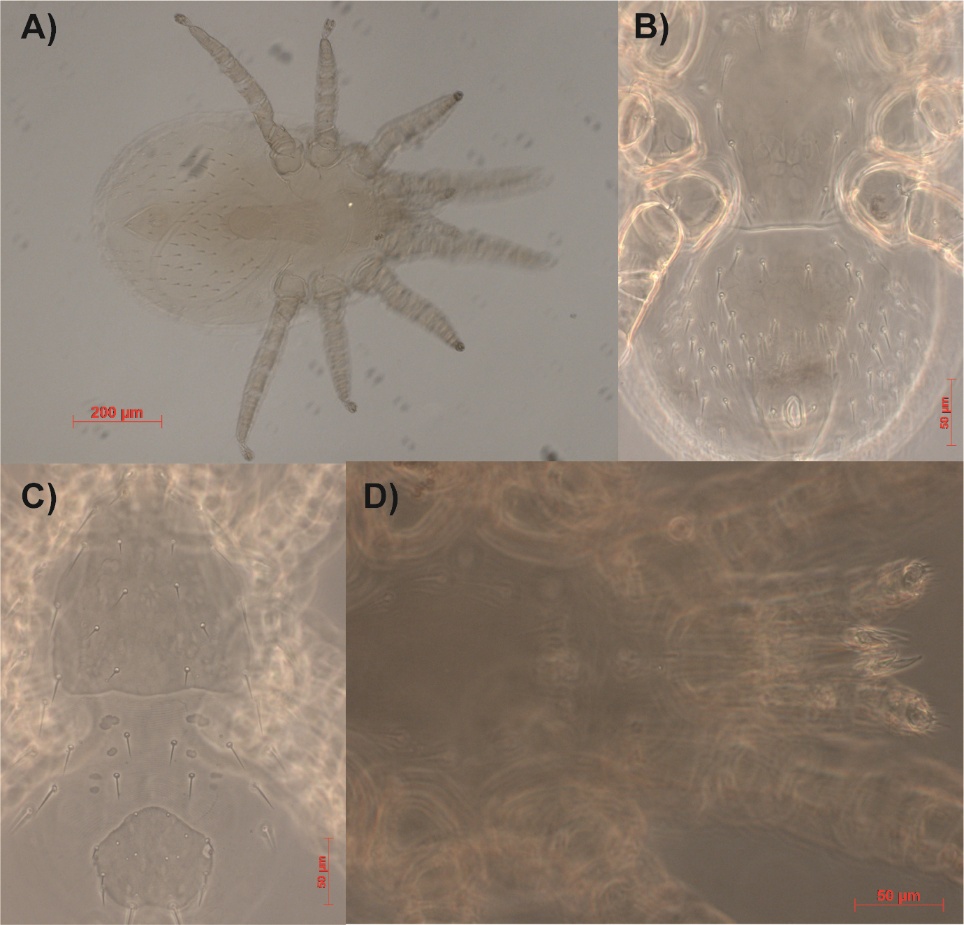  A) Ventral view (female). B) Holoventral plate (male). C) Dorsal view (protonymph).  D) Spermadactyl (male). |
| Figure S5. *Macronyssus unidens* Radovsky, 1967  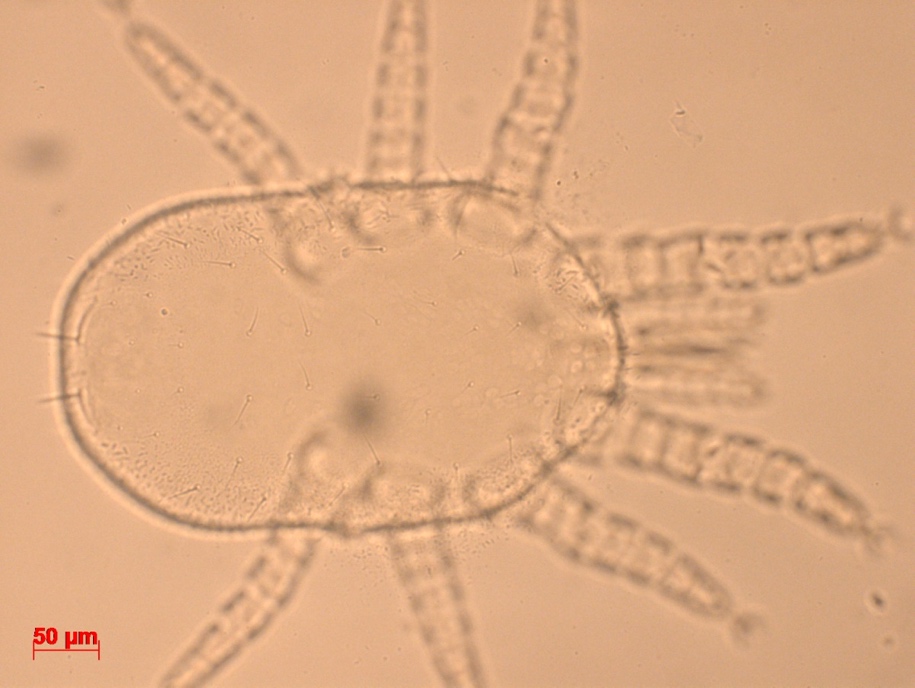  Idiosoma (dorsal view). |
| Figure S6. *Steatonyssus antrozoi* Radovsky & Furman, 1963  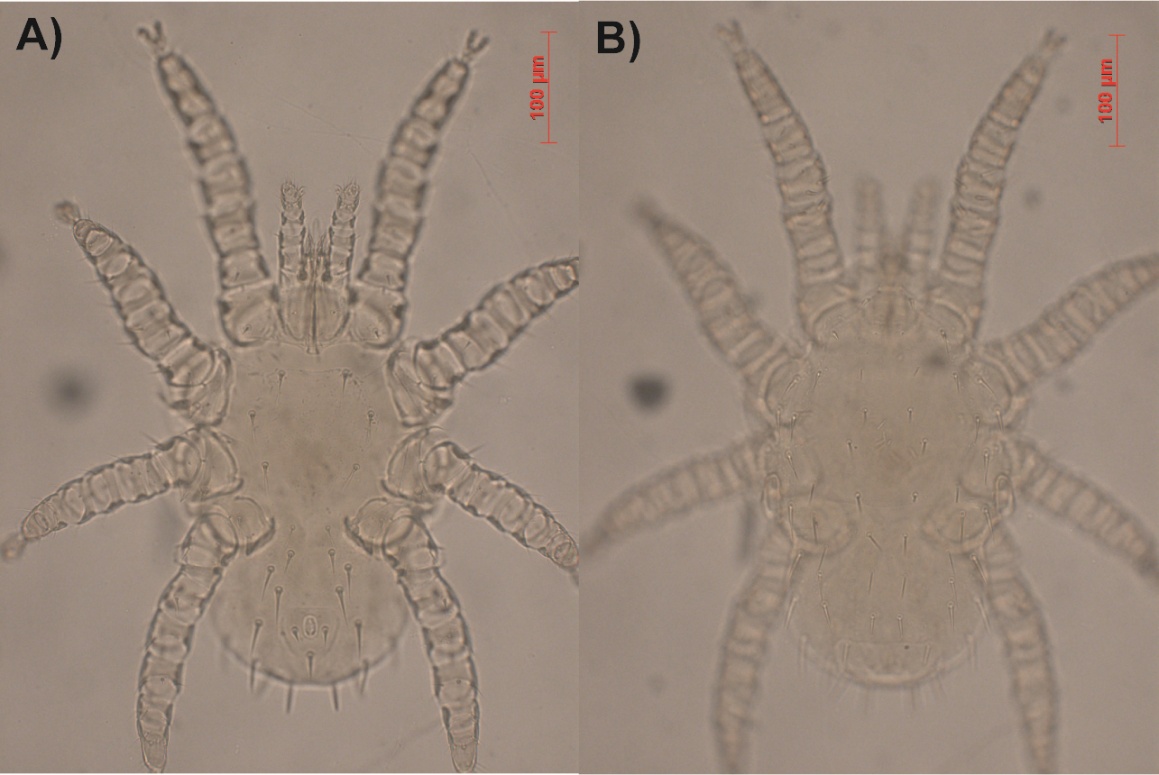  A) Dorsal view. B) Ventral view. |
| Figure S7. *Steatonyssus occidentalis* (Ewing, 1933)  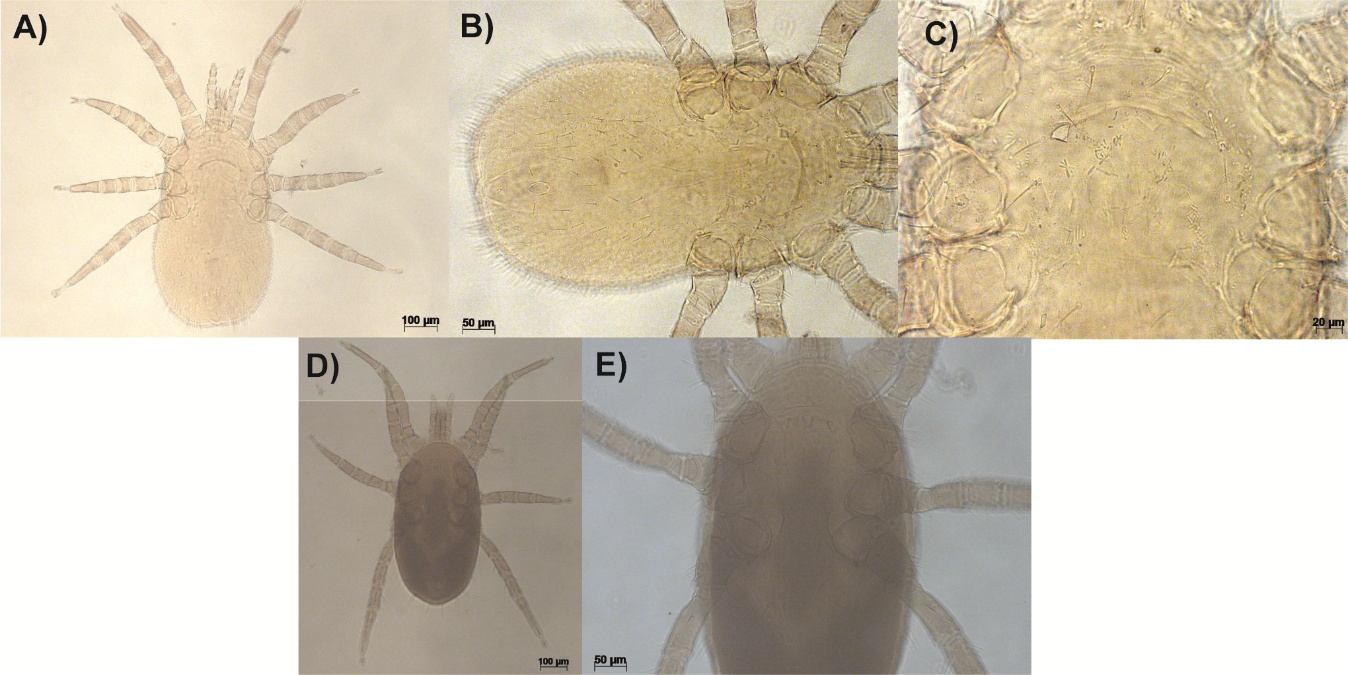   1. Female. B) Sternal, genital and anal plates. C) Zoom of sternal and anal plates (part). D) Male. E) Genital pore and holoventral plate. |
| Spinturnicidae Oudemans, 1902 |
| Figure S8. *Periglischrus paracaligus* Herrin & Tipton, 1975  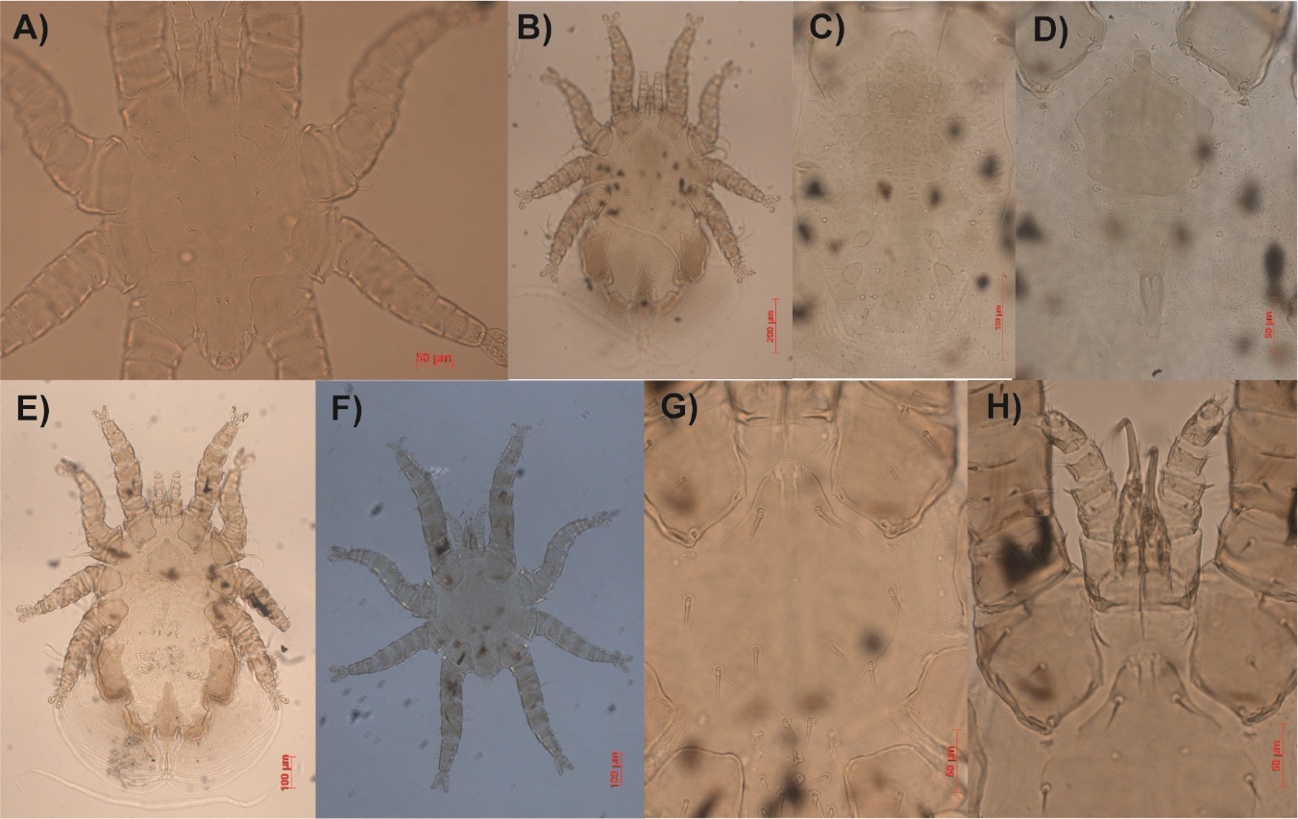  A) Protonymph. B) Female. C) Dorsal plate. D) Sternal and genital plates. E) Pregnant female. F) Male. G) Sternogenital plate. H) Spermadactyl. |
|  |
| Figure S9. *Spinturnix mexicana* Rudnick, 1960  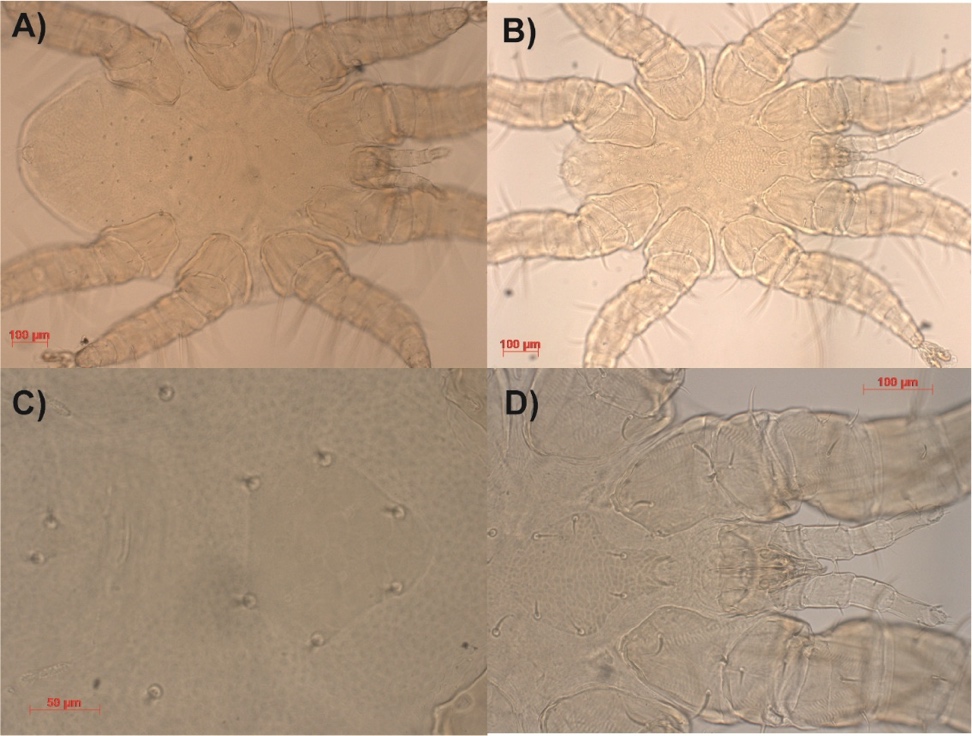  A) Female. B) Male. C) Sternal and genital plates (female). D) Sternogenital plate and gnatostoma (male). |
| Trombiculidae Ewing, 1929 |
| Figure S10. Trombiculidae Gen. sp. 1  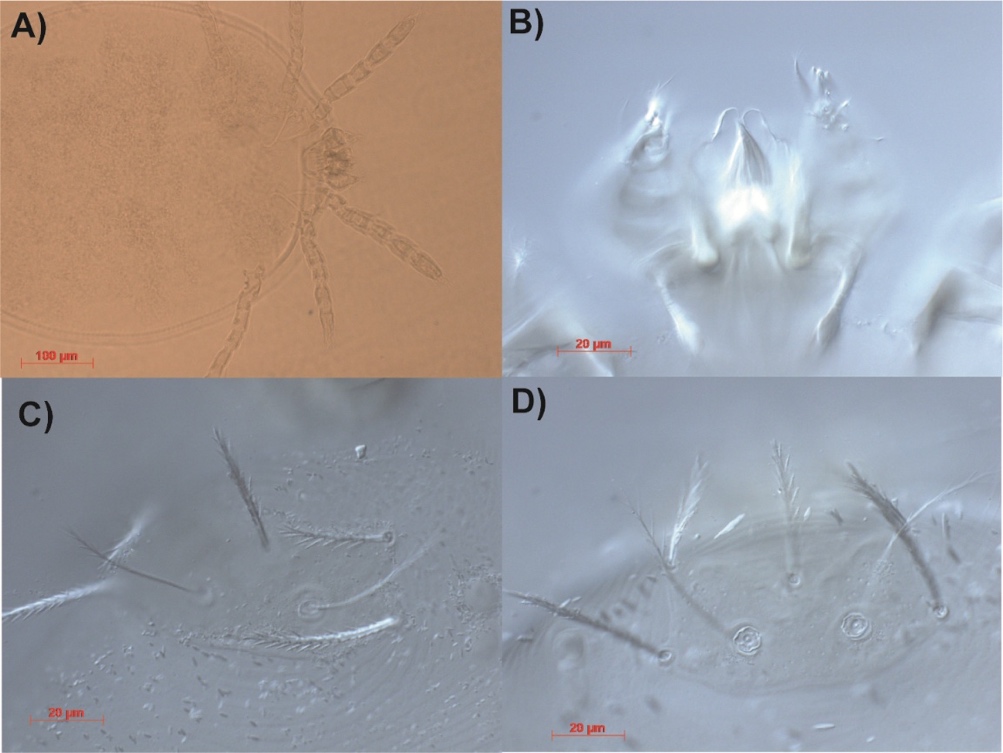  A) Idiosoma (larvae). B) Gnatosoma. C) and D) Prodorsal plate. |
|  |
| Figure S11. *Euschoengastia* sp.  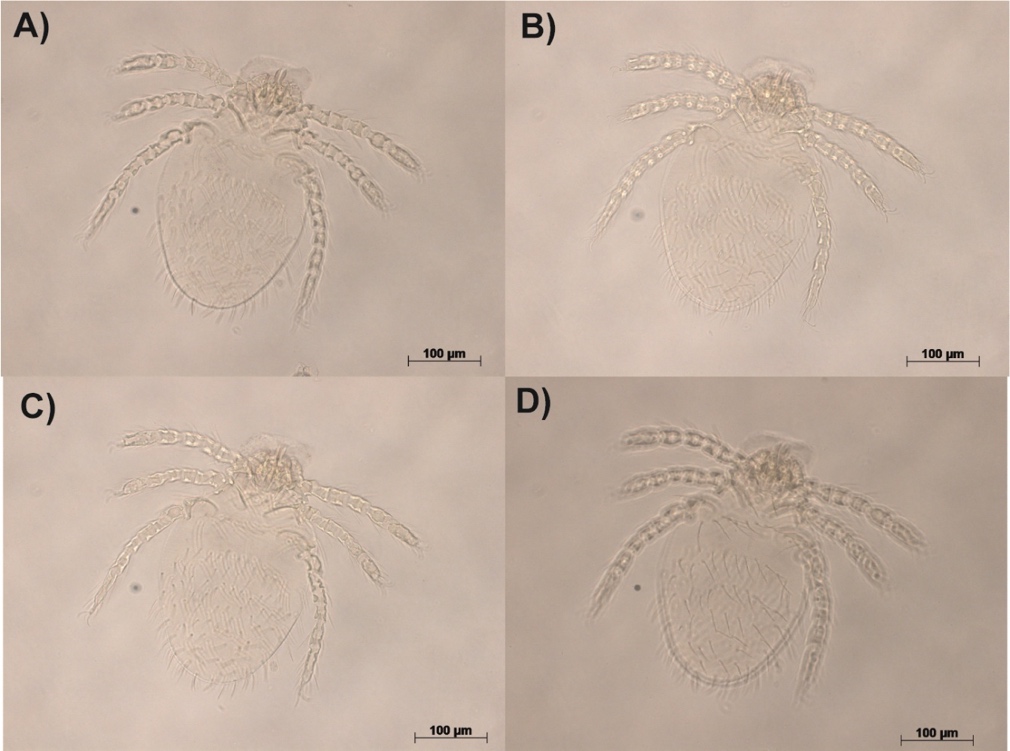  A) Gnatosoma. B). Dorsal plate C). Legs. D). Setae. |
| Figure S12. *Loomisia* sp.  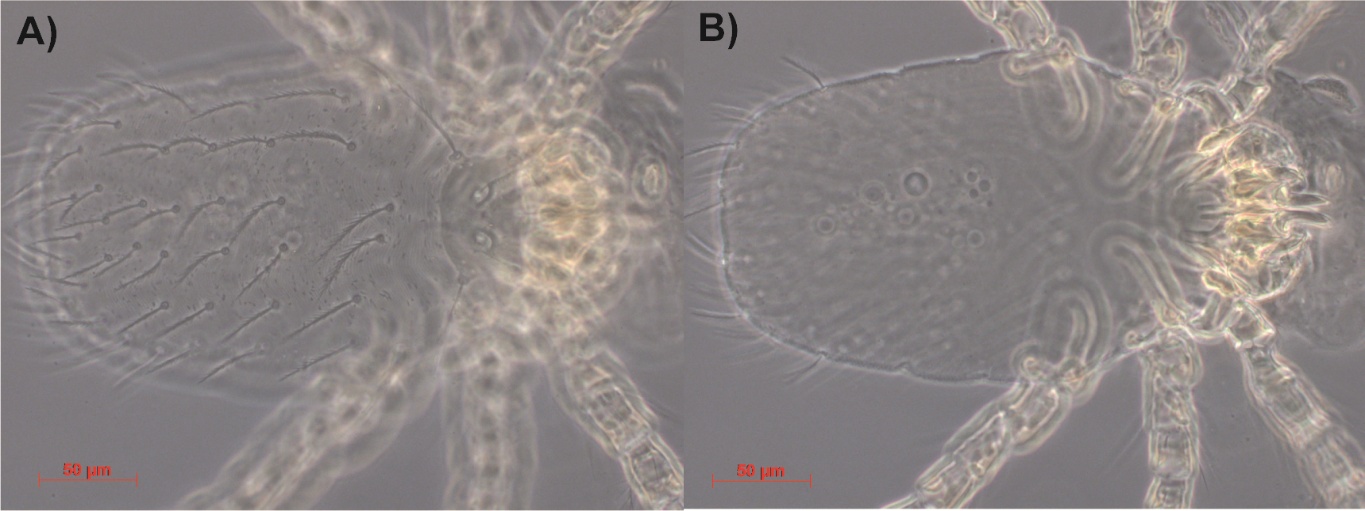  A) Dorsal view. B) Ventral view. |
|  |
| Leeuwenhoekiidae Womersley,1945 |
| Figure S13. *Albeckia albecki* Vercammen-Grandjean & Watkins, 1966  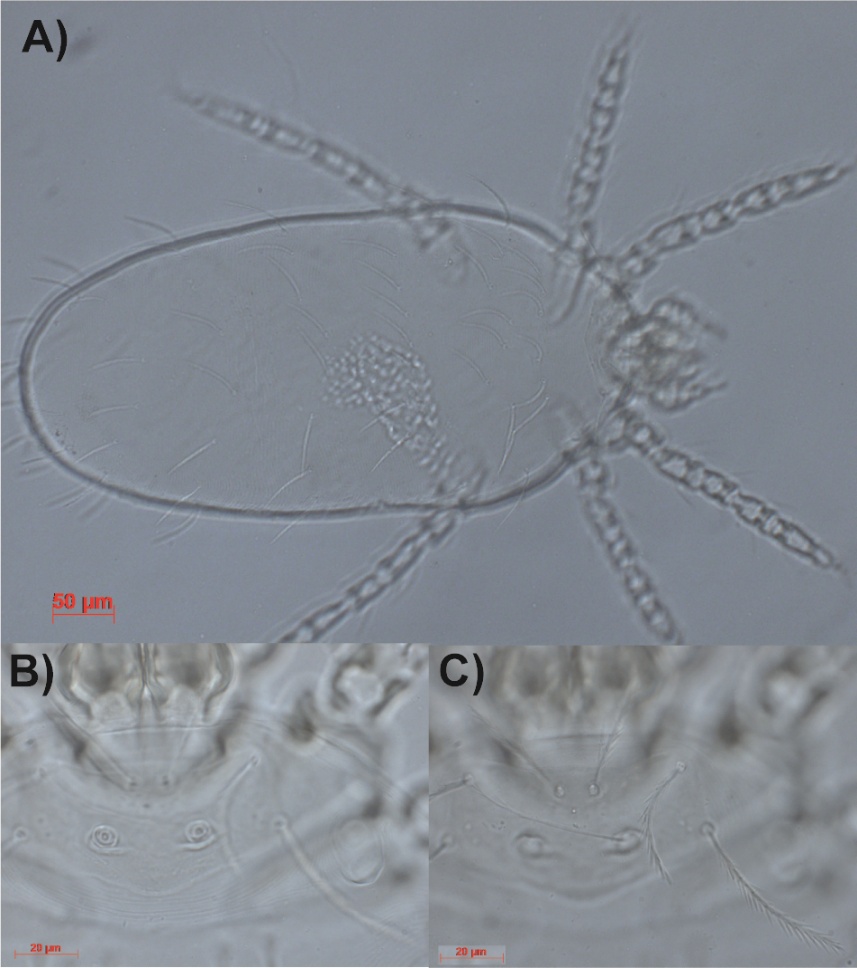  A) Idiosoma. B) Eyes. C) Setae. |
| Myobiidae Mégnin, 1877 |
| Figure S14. *Acanthophthirius caudatus eptesicus* Fain & Whitaker 1976  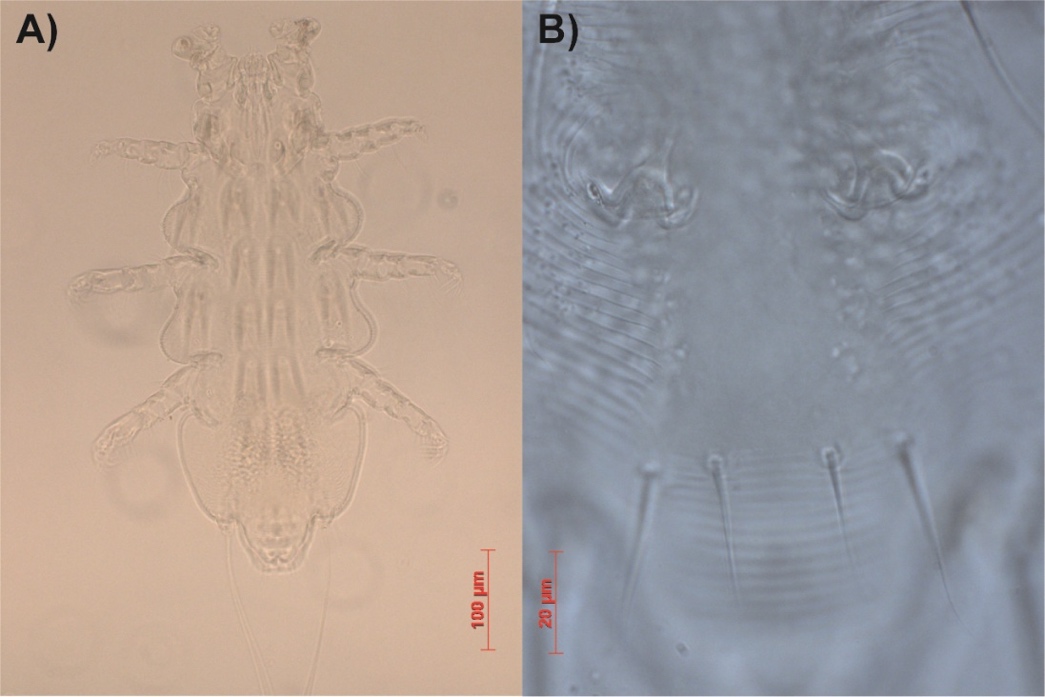  A) (Inter)dorsal view. B) Detailed photo of the opisthostome and opisthogastric sclerites. |
| Figure S15. *Acanthophthirius* sp. 1  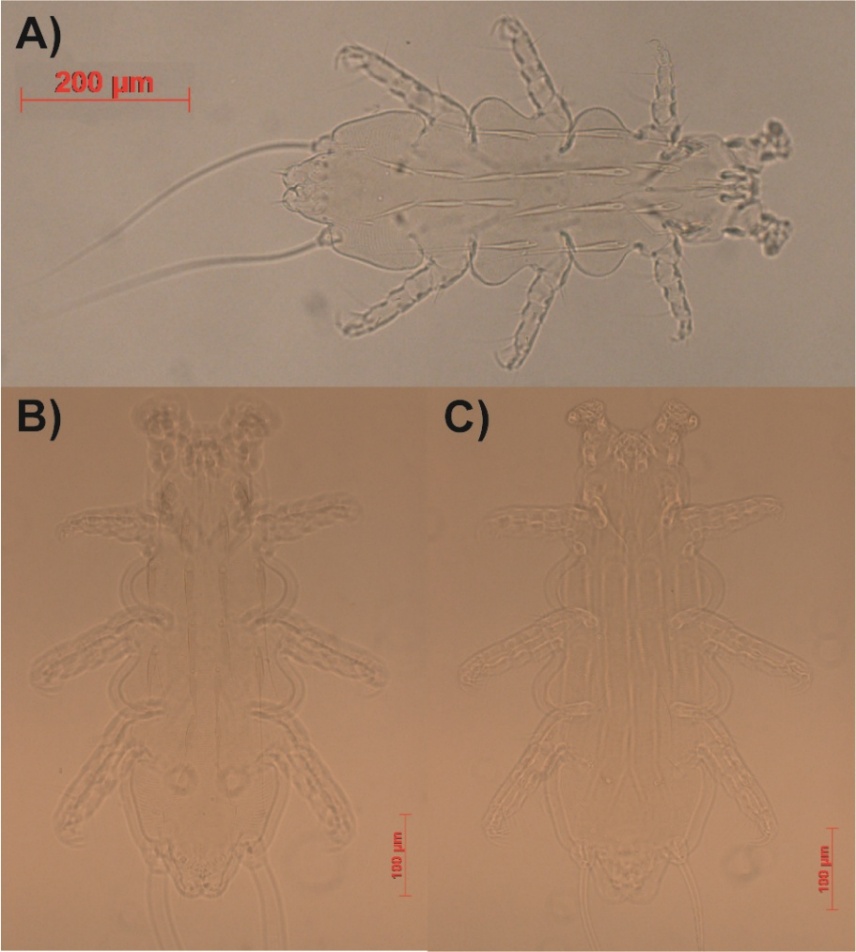  A) Idiosoma (female). B) Dorsal view. C) Ventral view. |
| Figure S16. *Acanthophthirius* sp. 2  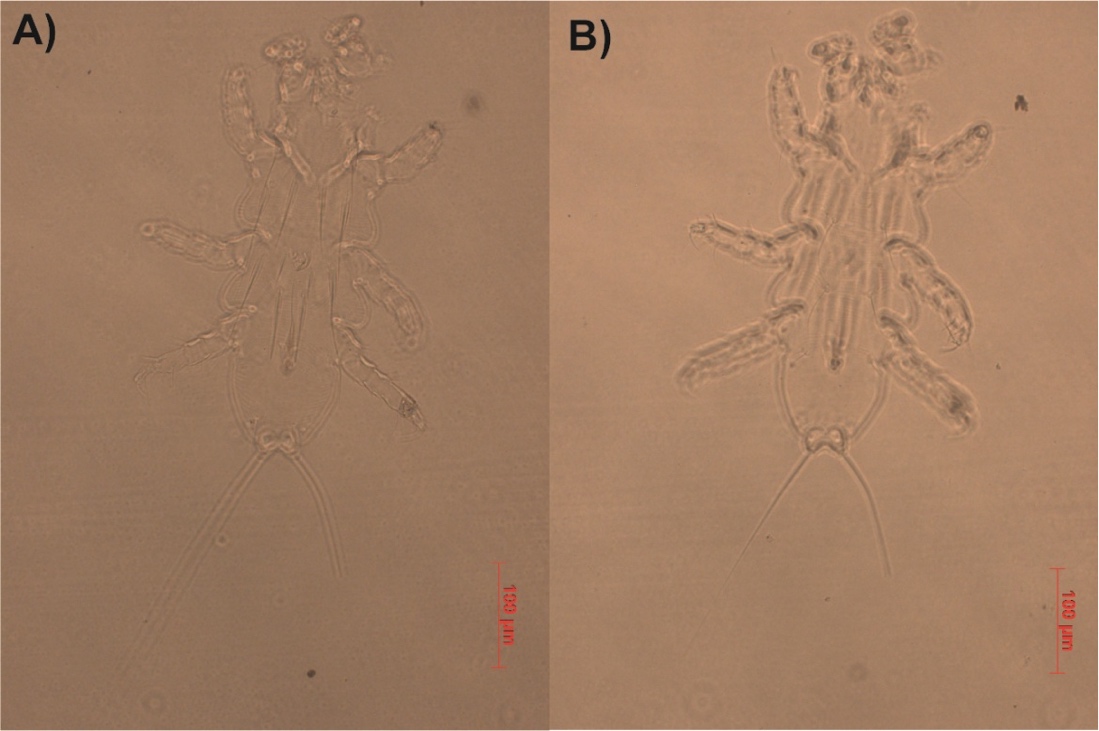  A) Dorsal view. B) Ventral view. |
| **Diptera** |
| Nycteribiidae Samouelle, 1819 |
| Figure S17. *Basilia antrozoi* (Townsend, 1893)  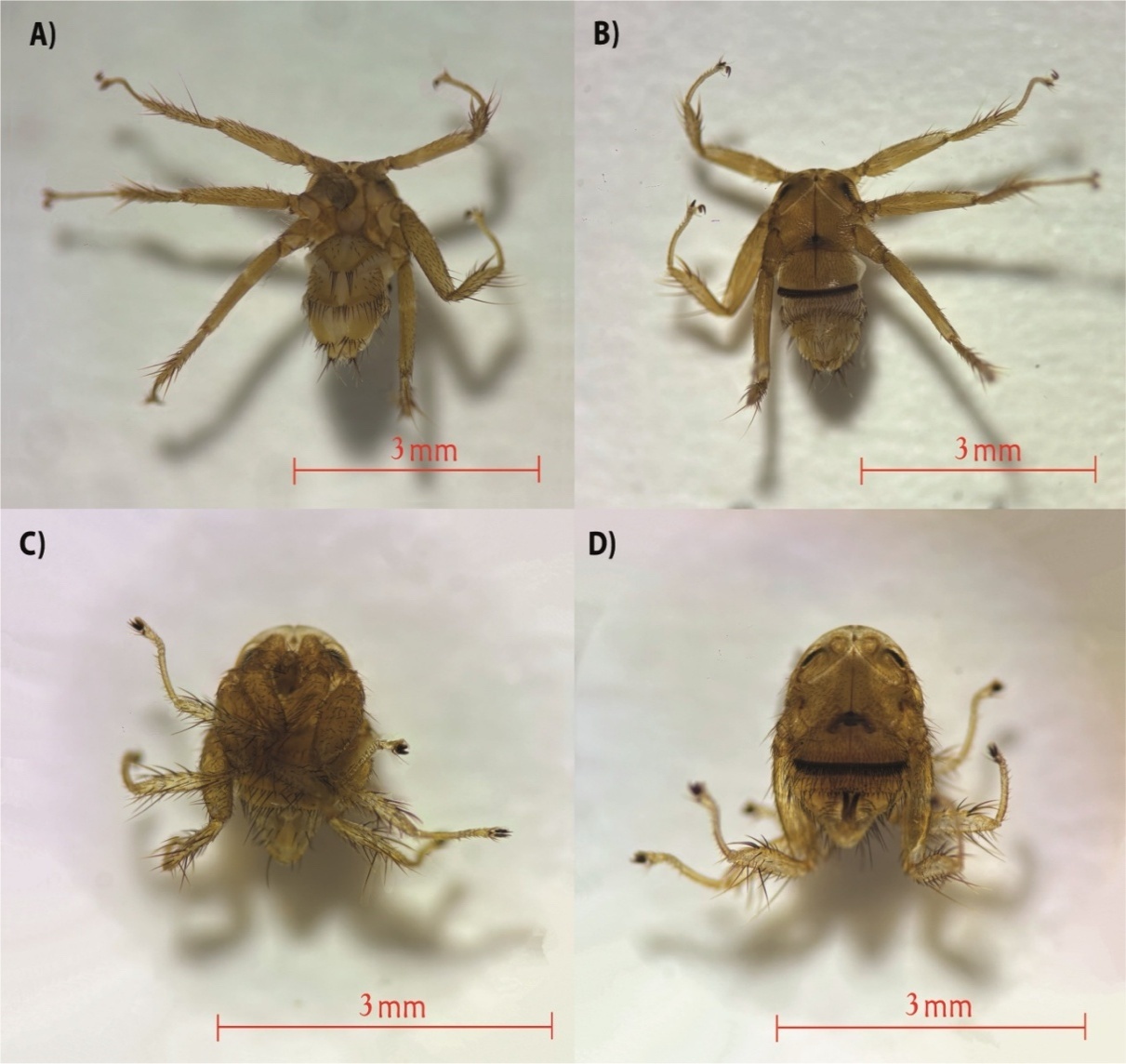  A) Dorsal view (female). B) Ventral view (female). C) Dorsal view (male). D) Ventral view (male). |
| Figure S18. *Basilia corynorhini* (Ferris, 1916)  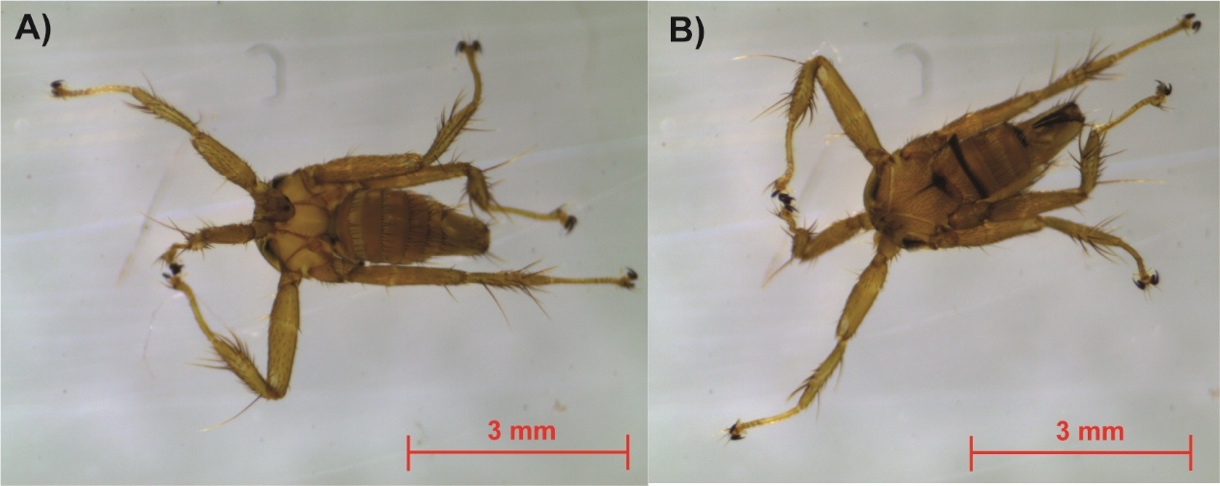  Male. A) Dorsal view. B) Ventral view. |
| Figure S19. *Basilia forcipata* Ferris, 1924  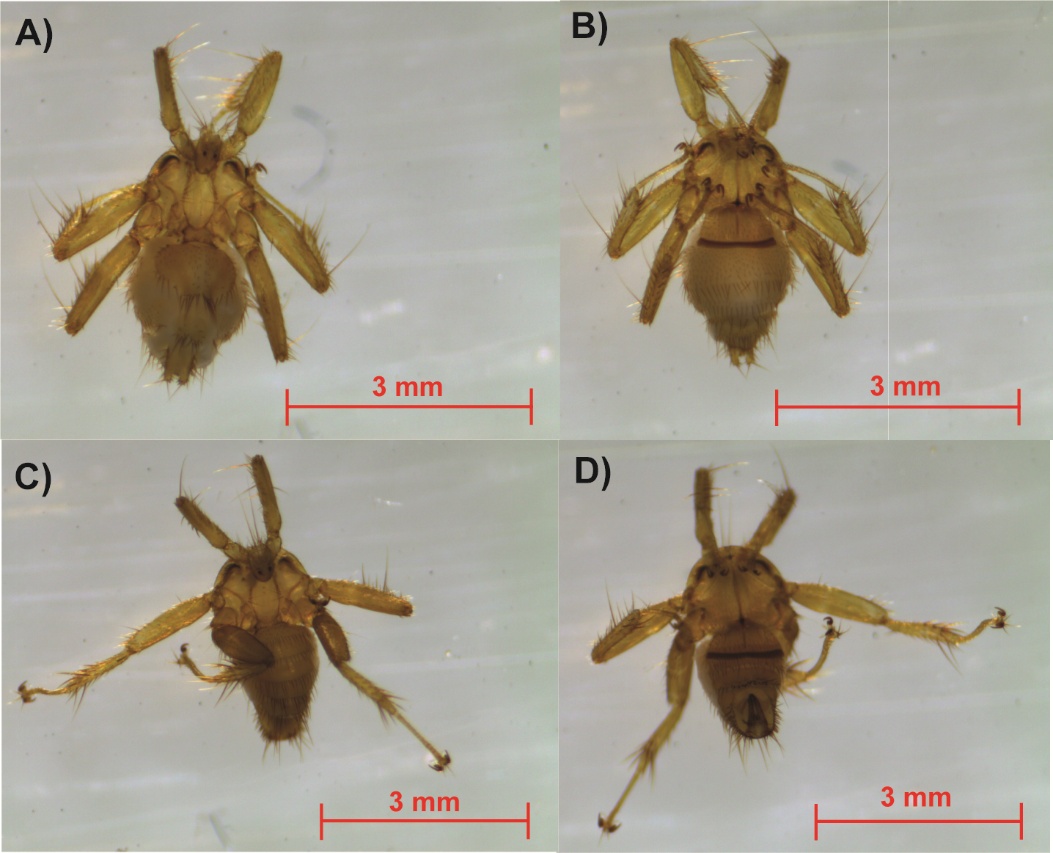  A) Dorsal view (female; extended abdomen possibly with eggs). B) Ventral view (female). C) Dorsal view (male). D) Ventral view (male). |
| Figure S20. *Basilia pizonychus* Scott, 1939  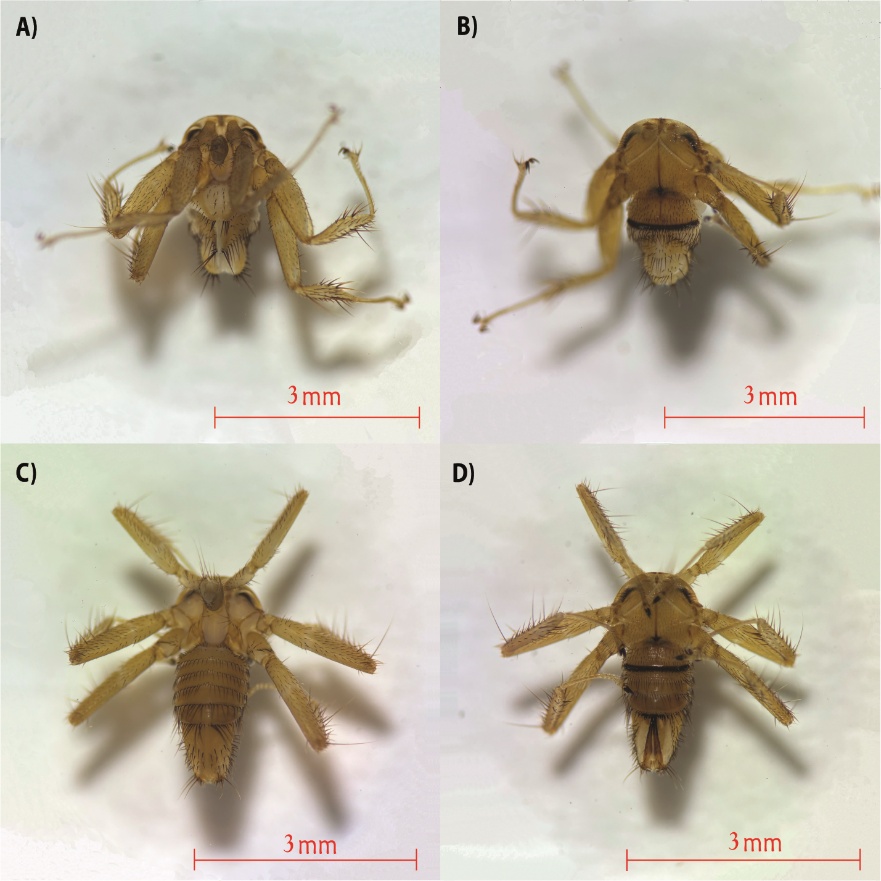  A) Dorsal view (female). B) Ventral view (female). C) Dorsal view (male). D) Ventral view (male). |
| Streblidae Kolenati, 1863 |
| Figure S21. *Nycterophilia coxata* Ferris, 1916  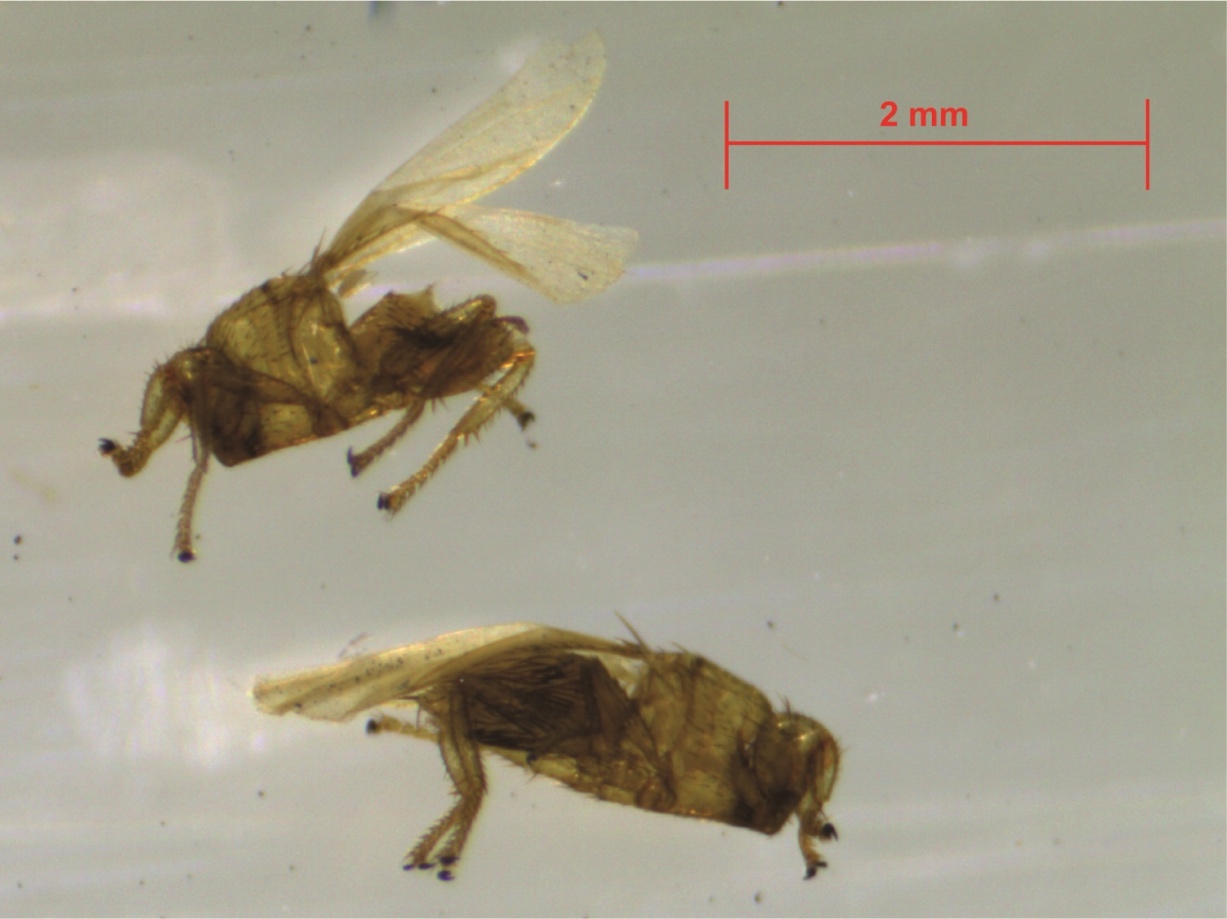  Lateral view of male (upper) and female (lower). |
| Figure S22. *Trichobius sphaeronotus* Jobling, 1939  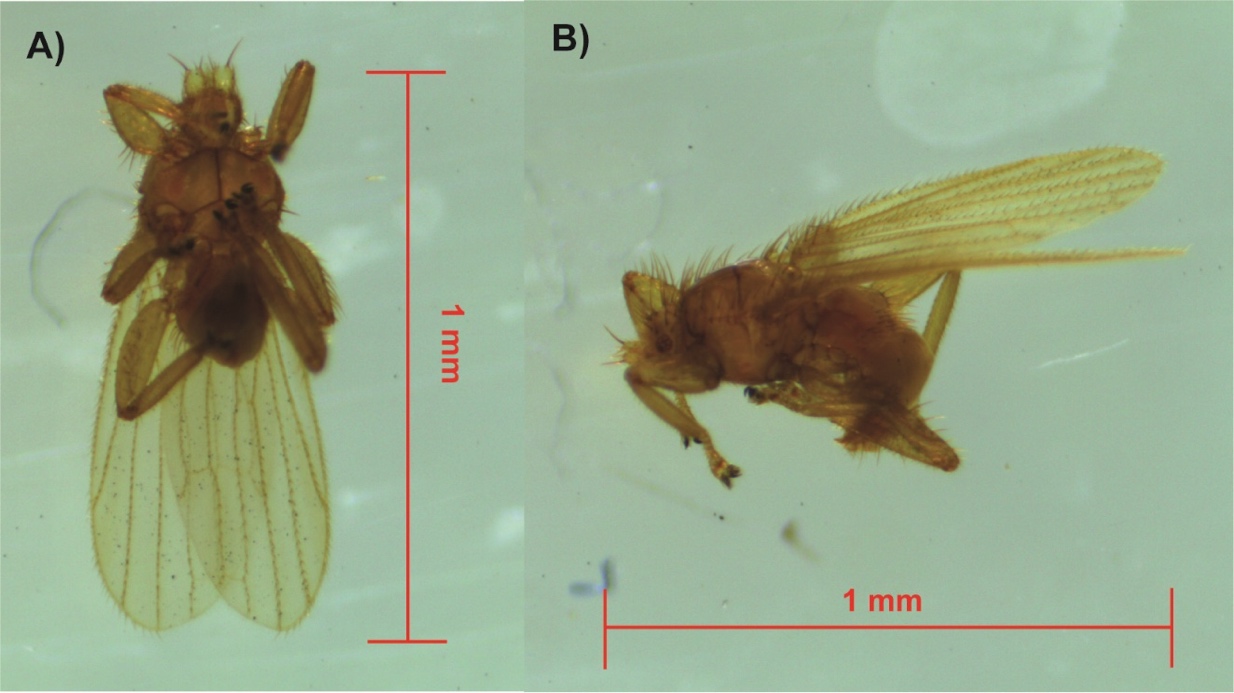  Male. A) Ventral view. B) Lateral view. |
| **Hemiptera** |
| Cimicidae Latreille, 1802 |
| Figure S23. *Cimex pilosellus* (Horvath, 1910)  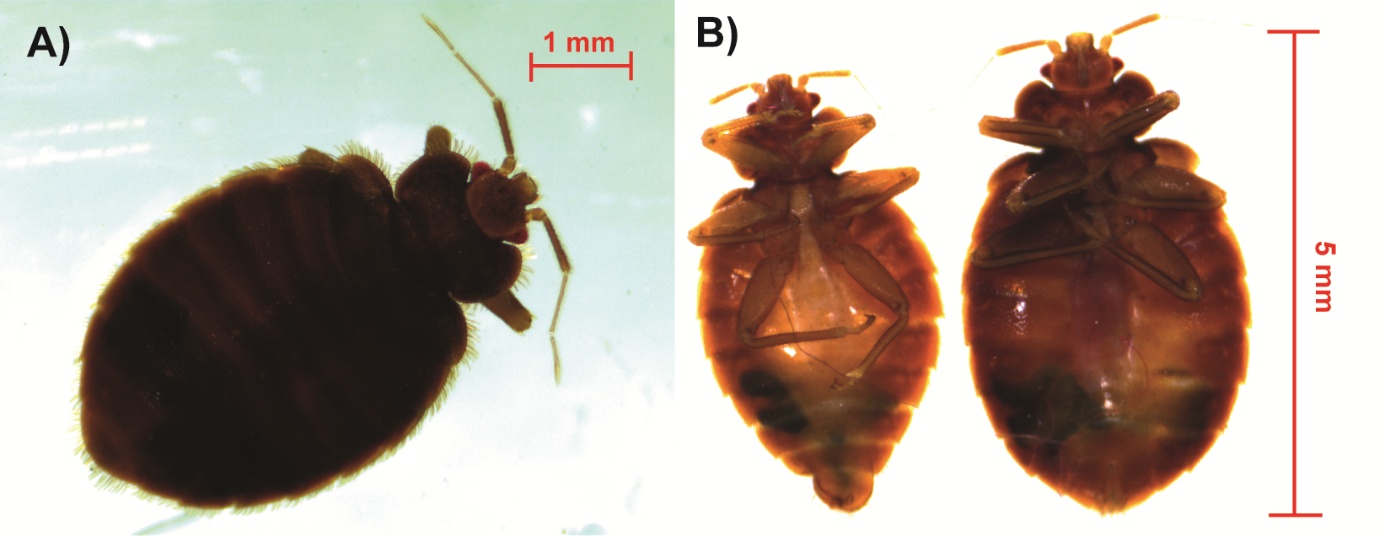  A) Dorsal view (female). B) Ventral view of male (left) and female (right). |
